# Supplementary material for: Testicular macrophages are recruited during a narrow fetal time window and promote organ-specific developmental functions
Source: Nat Commun. 2023 Mar 15;14:1439. doi: 10.1038/s41467-023-37199-0 (PMC10017703; doi:10.1038/s41467-023-37199-0)
Supplement: Supplementary file 1 — Supplementary Information [file 41467_2023_37199_MOESM1_ESM.pdf]

Supplementary Information

Testicular macrophages are recruited during a narrow fetal time window and promote organ-specific developmental functions

Xiaowei Gu, Anna Heinrich, Shu-Yun Li, and Tony DeFalco

Correspondence: tony.defalco@cchmc.org (T.D.)

This PDF file includes:

Supplementary Figs. 1-18

Supplementary Tables 1-2

Supplementary References

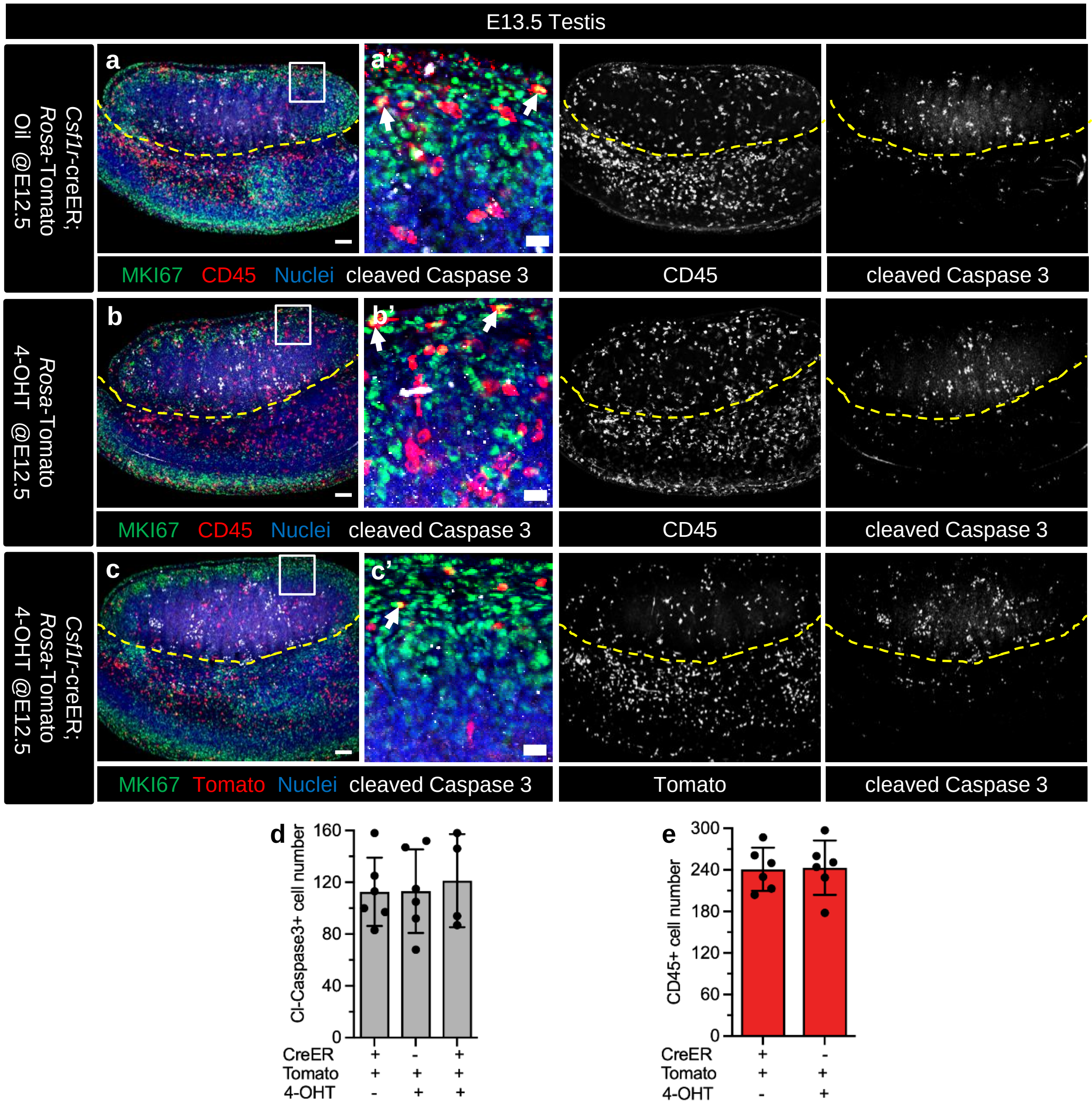

Supplementary Figure 1. Tamoxifen administration has no rapid, acute effect on apoptosis or immune cell number in early fetal testes. (a-c) Representative images of E13.5 fetal testes from *Csf1r*-creER; *Rosa*-Tomato embryos exposed to vehicle only (ethanol + oil) at E12.5 (a), CreER-negative; *Rosa*-Tomato embryos exposed to 4-OHT at E12.5 (b), and *Csf1r*-creER; *Rosa*-Tomato embryos exposed to 4-OHT at E12.5 (c). In all supplementary figures in this study, prime figures (e.g., a' relative to a) are higher-magnification images of the boxed region(s) in the image to their left. Dashed lines indicate gonad-mesonephros boundary. Arrows indicate MKI67+CD45+ (a, b) or MKI67+Tomato+ (c) cells. Thin scale bar, 100 µm; thick scale bar, 25 µm. (d, e) Graphs showing quantification of cleaved-Caspase-3-positive (d) or CD45+ (e) cells per gonadal optical section from E13.5 fetal testes of various *Csf1r*-creER; *Rosa*-Tomato genotypes and treatments listed below the graph (same as images in a-c) (n=6 independent *Csf1r*-creER; *Rosa*-Tomato gonads exposed to vehicle; n=6 independent creER-negative; *Rosa*-Tomato gonads exposed to 4-OHT; n=4 independent *Csf1r*-creER; *Rosa*-Tomato gonads exposed to 4-OHT). Data are shown as mean +/- SD. All comparisons are not significant (P>0.05; two-tailed Student's t-test). Exact P values are provided in the Source Data file.

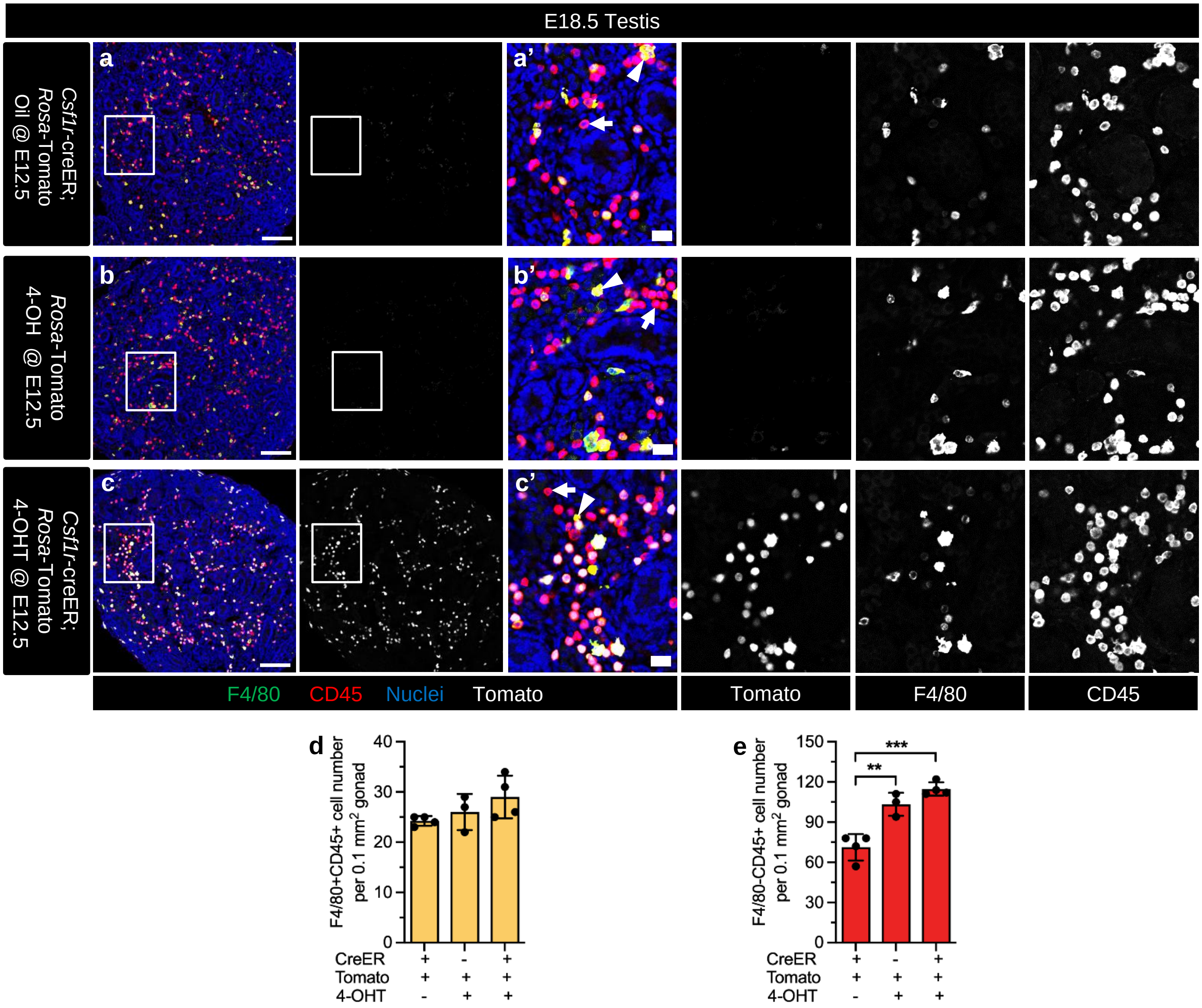

Supplementary Figure 2. *Rosa*-Tomato reporter allele does not show spurious activation in absence of tamoxifen administration, but tamoxifen treatment results in longer-term increase in fetal testicular F4/80-CD45+ immune cells. (a-c) Representative images of E18.5 fetal testes from *Csf1r*-creER; *Rosa*-Tomato embryos exposed to vehicle only (ethanol + oil) at E12.5 (a), CreER-negative; *Rosa*-Tomato embryos exposed to 4-OHT at E12.5 (b), and *Csf1r*-creER; *Rosa*-Tomato mice exposed to 4-OHT at E12.5 (c). Arrows indicate F4/80-CD45+ and arrowheads indicate F4/80+CD45+ cells. Thin scale bar, 100 µm; thick scale bar, 25 µm. (d, e) Graphs showing quantification of F4/80+CD45+ (d) or F4/80-CD45+ (e) cells per unit area of gonad from E18.5 fetal testes of various *Csf1r*-creER; *Rosa*-Tomato genotypes and treatments listed below the graph (same as images in a-c) (*n*=4 independent *Csf1r*-creER; *Rosa*-Tomato gonads exposed to vehicle; *n*=3 independent creER- negative; *Rosa*-Tomato gonads exposed to 4-OHT; *n*=4 independent *Csf1r*-creER; *Rosa*-Tomato gonads exposed to 4-OHT). Data are shown as mean +/- SD. ***P*<0.01; ****P*<0.001 (two-tailed Student's *t*-test). Exact *P* values are provided in the Source Data file.

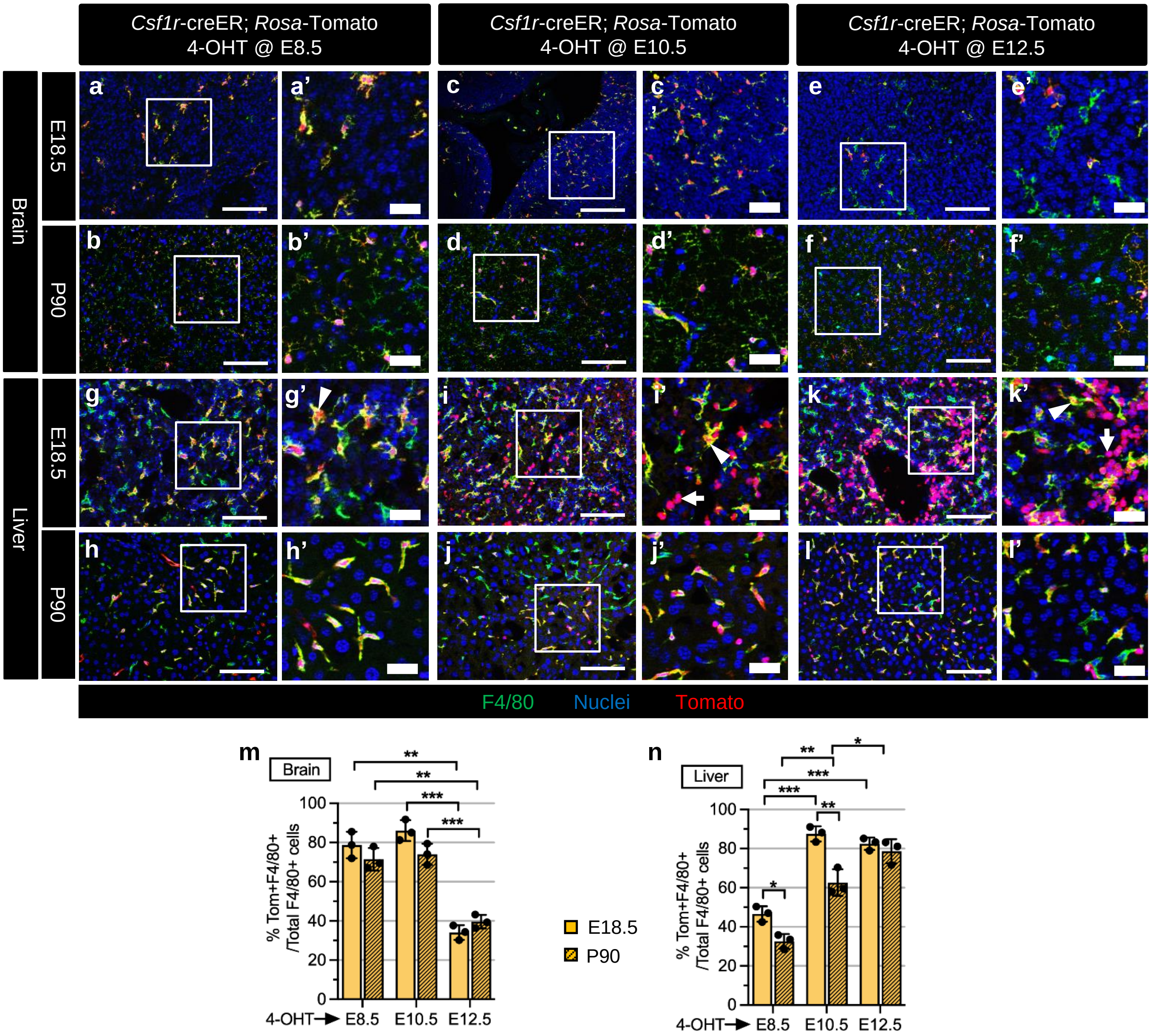

Supplementary Figure 3. YS-derived EMPs contribute to brain microglia and liver Kupffer cells. (a-l) Representative images (*n*=3) of brain (a-f) and liver (g-l) at E18.5 (a, c, e, g, i, k) or P90 (b, d, f, h, j , l) from *Csf1r*-creER; *Rosa*-Tomato embryos exposed to 4-OHT at E8.5 (a, b, g, h), E10.5 (c, d, i ,j), or E12.5 (e, f, k ,l). Arrowheads and arrows in g', i', and k' denote Tomato-expressing F4/80+ macrophages and Tomato-expressing F4/80-negative cells, respectively. Thin scale bar, 100 µm; thick scale bar, 25 µm. (m, n) Graphs showing quantification (*n*=3) of percent Tomato-expressing F4/80+ macrophages in E18.5 or P90 brain (m) or liver (n) from *Csf1r*-creER; *Rosa*-Tomato mice induced with 4-OHT at various embryonic stages. Data are shown as mean +/- SD. **P*<0.05; ***P*<0.01; ****P*<0.001 (two-tailed Student's *t*-test). Exact *P* values are provided in the Source Data file.

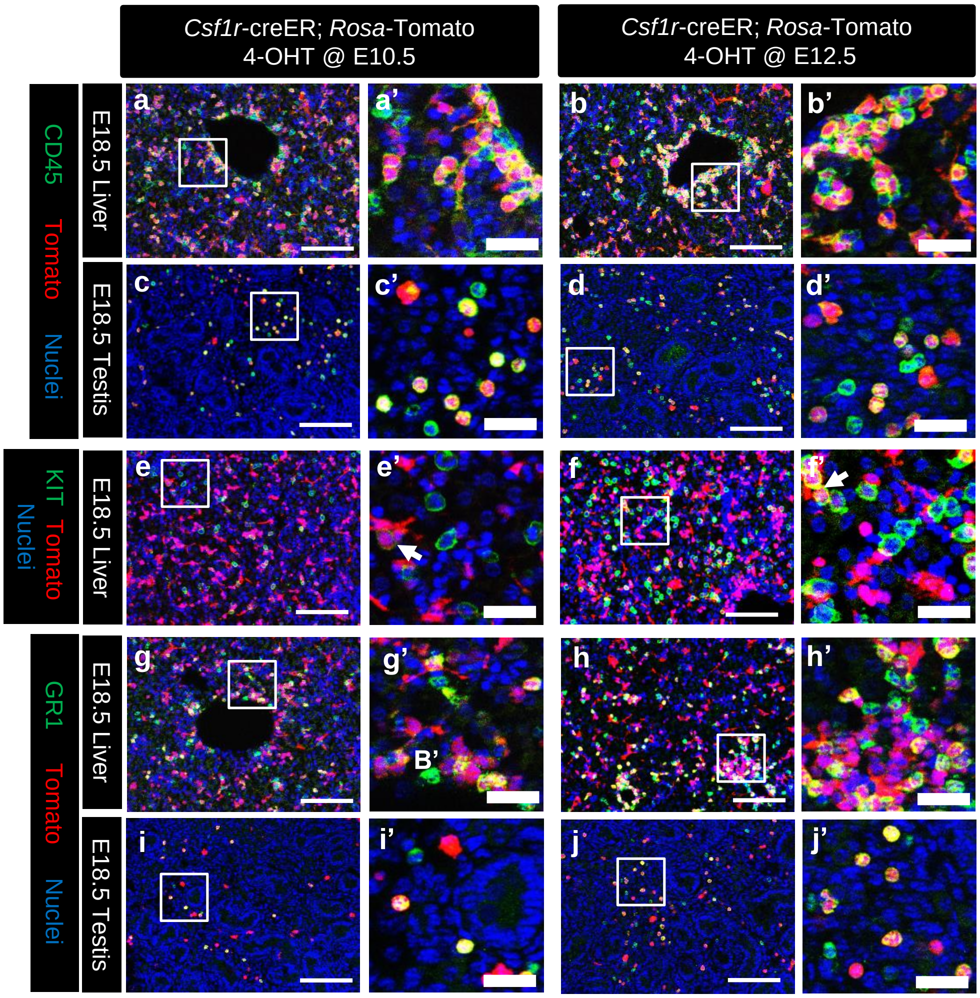

Supplementary Figure 4. Fetal *Csf1r*+ definitive progenitors give rise to myeloid cells. (a-j) Representative images (*n*=3) of E18.5 liver (a, b, e-h) and testis (c, d, i, j) from *Csf1r*-creER; *Rosa*-Tomato embryos exposed to 4-OHT at E10.5 (a, c, e, g, i) or E12.5 (b, d, f, h, l) stained for various hematopoietic markers such as CD45 (pan-immune marker), KIT (HSC marker), and GR1 (neutrophil/monocyte marker). Arrows in e' and f' denote Tomato-positive KIT+ cells. Thin scale bar, 100 µm; thick scale bar, 25 µm.

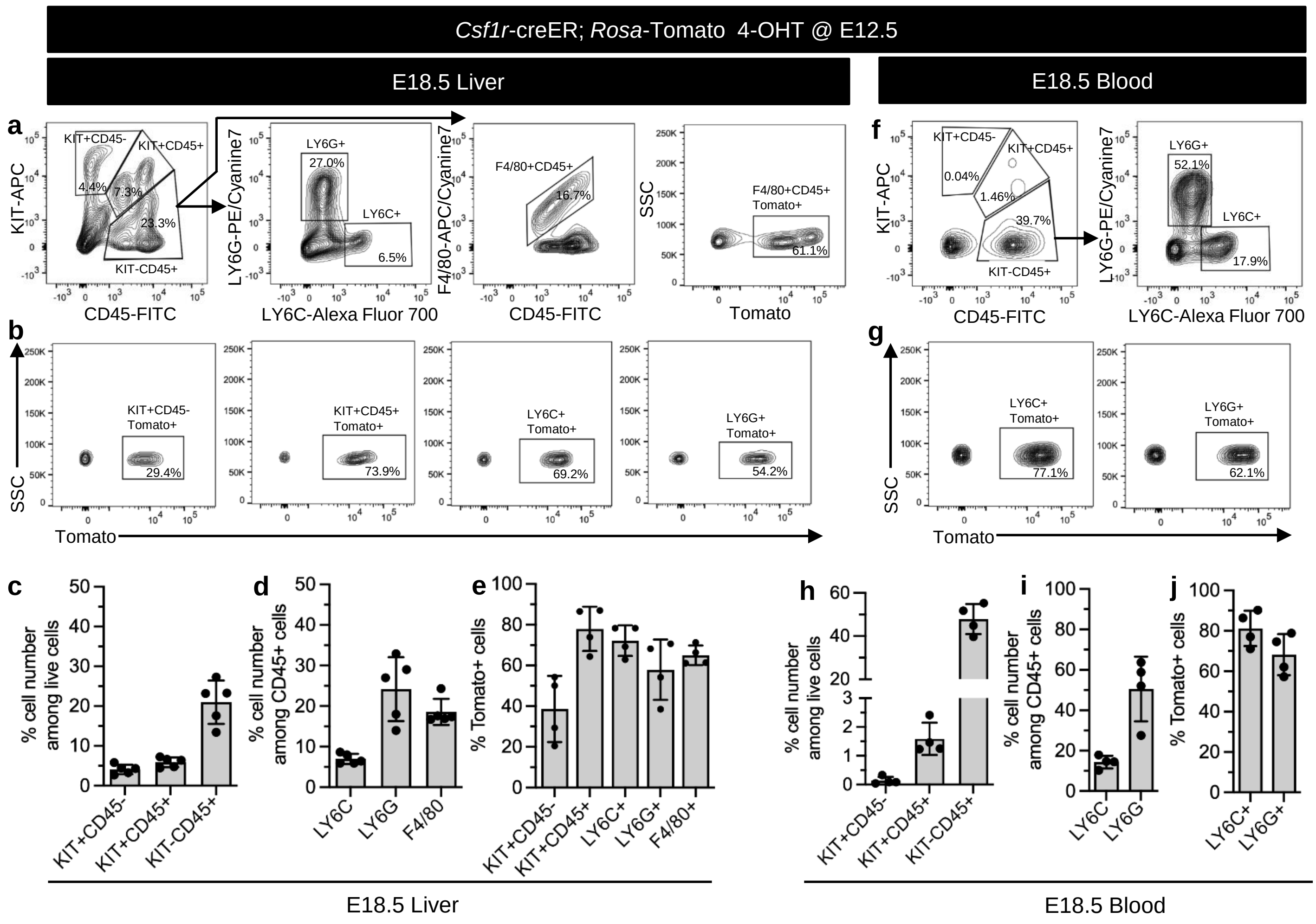

Supplementary Figure 5. *Csf1r*-CreER-mediated lineage tracing labels monocytes and neutrophils in fetal liver and blood after the initial 4-OHT treatment window due at least partially to targeting of fetal liver HSCs. (a-j) Flow cytometric analyses of immune cell populations from fetal liver (a-e) and blood (f-j) of E18.5 *Csf1r*-creER; *Rosa*-Tomato embryos exposed to 4-OHT at E12.5. (a) Isolation of fetal liver KIT+ cells and CD45+ cells into LY6G+ neutrophil, LY6C+ monocyte, F4/80+ macrophage, and F4/80+Tomato+ cell populations. (b) Tomato expression in fetal liver KIT+CD45-, KIT+CD45+, LY6C+, and LY6G+ cell populations. (c-e) Graphs showing percent cell number among live cells (c), percent cell number among CD45+ cells (d), and percent Tomato+ cells (e) of various fetal liver cell populations (*n*=5 for c, d; *n*=4 for e). (f) Isolation of blood KIT+ cells and CD45+ cells into LY6G+ neutrophil and LY6C+ monocyte populations. (g) Tomato expression in blood LY6C+ and LY6G+ cell populations. (h-j) Graphs showing percent cell number among live cells (h), percent cell number among CD45+ cells (i), and percent Tomato+ cells (j) of various blood cell populations (*n*=4). Data are shown as mean +/- SD.

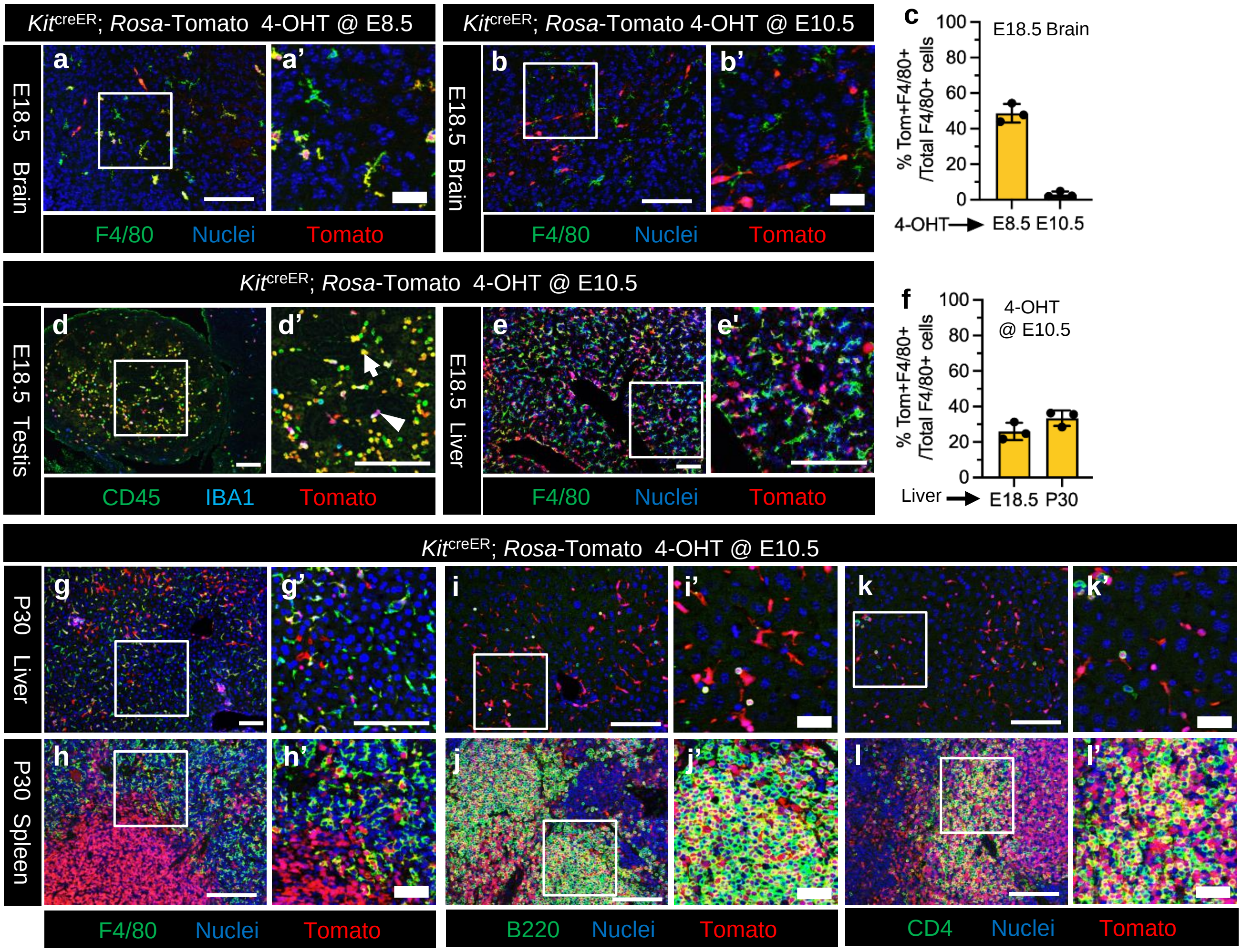

Supplementary Figure 6. Fetal AGM-derived HSCs give rise to multiple immune cell populations. Representative images (*n*=3) of E18.5 brain (a, b), E18.5 testis (d), E18.5 liver (e), P30 liver (g, i, k), and P30 spleen (h, j, l) from *Kit*^{creER}; *Rosa*-Tomato mice exposed to 4-OHT at E8.5 (a) or E10.5 (b, d, e, g-l) stained for various immune cell populations. Arrowhead in d' denotes a Tomato-expressing IBA1+ macrophage and arrow in d' denotes a Tomato-expressing CD45+ IBA1-negative cell. Thin scale bar, 100 µm; thick scale bar, 25 µm. (c) Graphs showing quantification (*n*=3) of percent Tomato-expressing F4/80+ macrophages in fetal brain from E18.5 *Kit*^{creER}; *Rosa*-Tomato embryos exposed to 4-OHT at E8.5 or E10.5. (f) Graphs showing quantification (*n*=3) of percent Tomato-expressing F4/80+ macrophages in liver from E18.5 or P30 *Kit*^{creER}; *Rosa*-Tomato mice exposed to 4-OHT at E10.5. Data are shown as mean +/- SD.

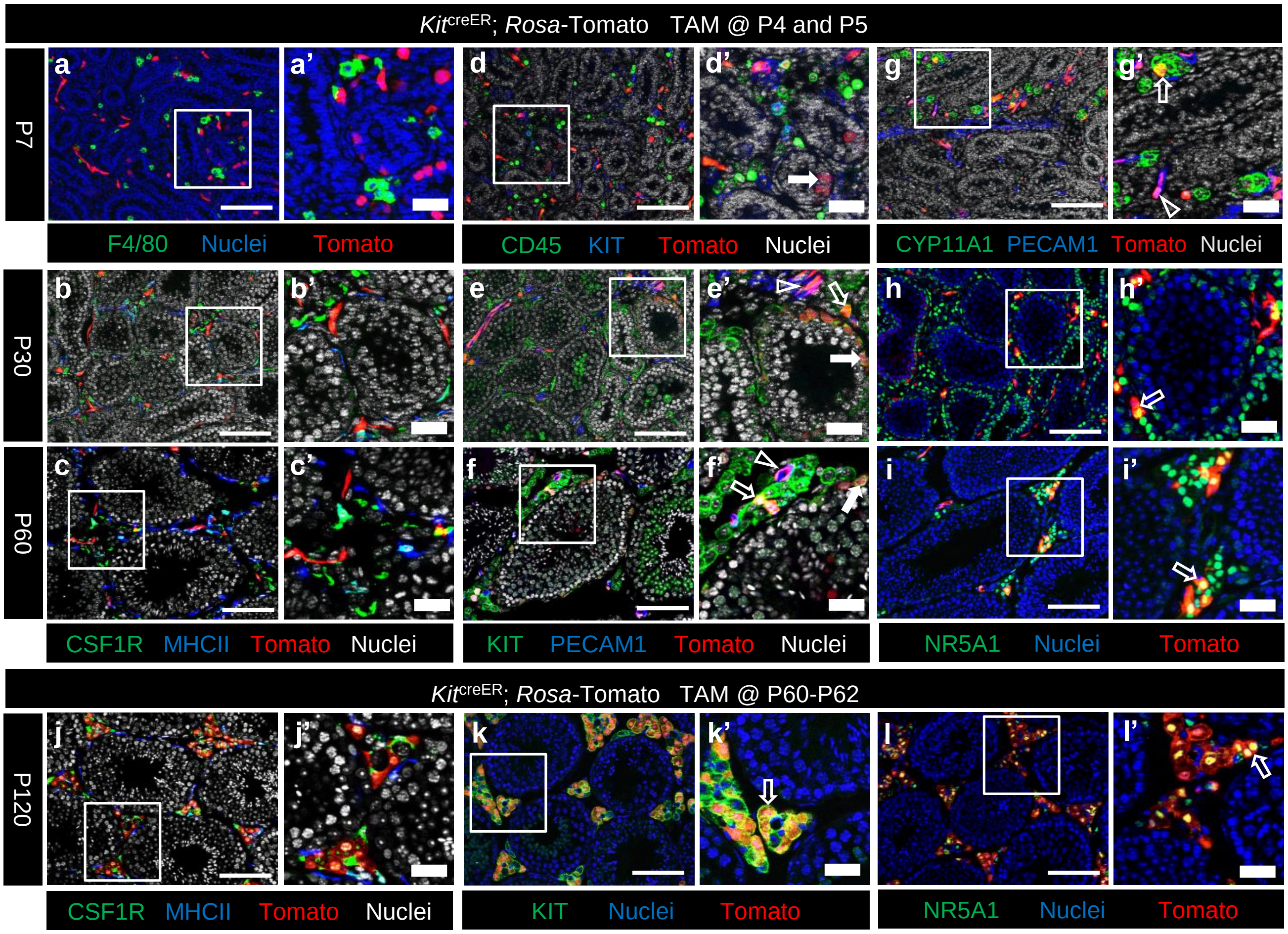

Supplementary Figure 7. Bone-marrow-derived HSCs do not contribute to testicular macrophages. (a-l) Representative images (n=3 independent testes) of testes from P7 (a, d, g), P30 (b, e, h), P60 (c, f, i), and P120 (j-l) *Kit*^{creER}; *Rosa*-Tomato mice exposed to TAM at P4 and P5 (a-i) or P60-P62 (j-l) stained for markers of various gonadal cell populations such as F4/80 (macrophages), CD45 (pan-immune cell marker), KIT (HSCs, differentiating spermatogonia, and adult Leydig cells), CYP11A1 (Leydig cells), PECAM1 (endothelial cells), CSF1R (adult interstitial macrophages), MHCII (adult peritubular macrophages), and NR5A1 (adult Leydig cells). Black arrows indicate Tomato+ Leydig cells; black arrowheads indicate Tomato+ endothelial cells; and white arrows indicate Tomato+ KIT-expressing differentiating spermatogonia. Thin scale bar, 100 μm; thick scale bar, 25 μm.

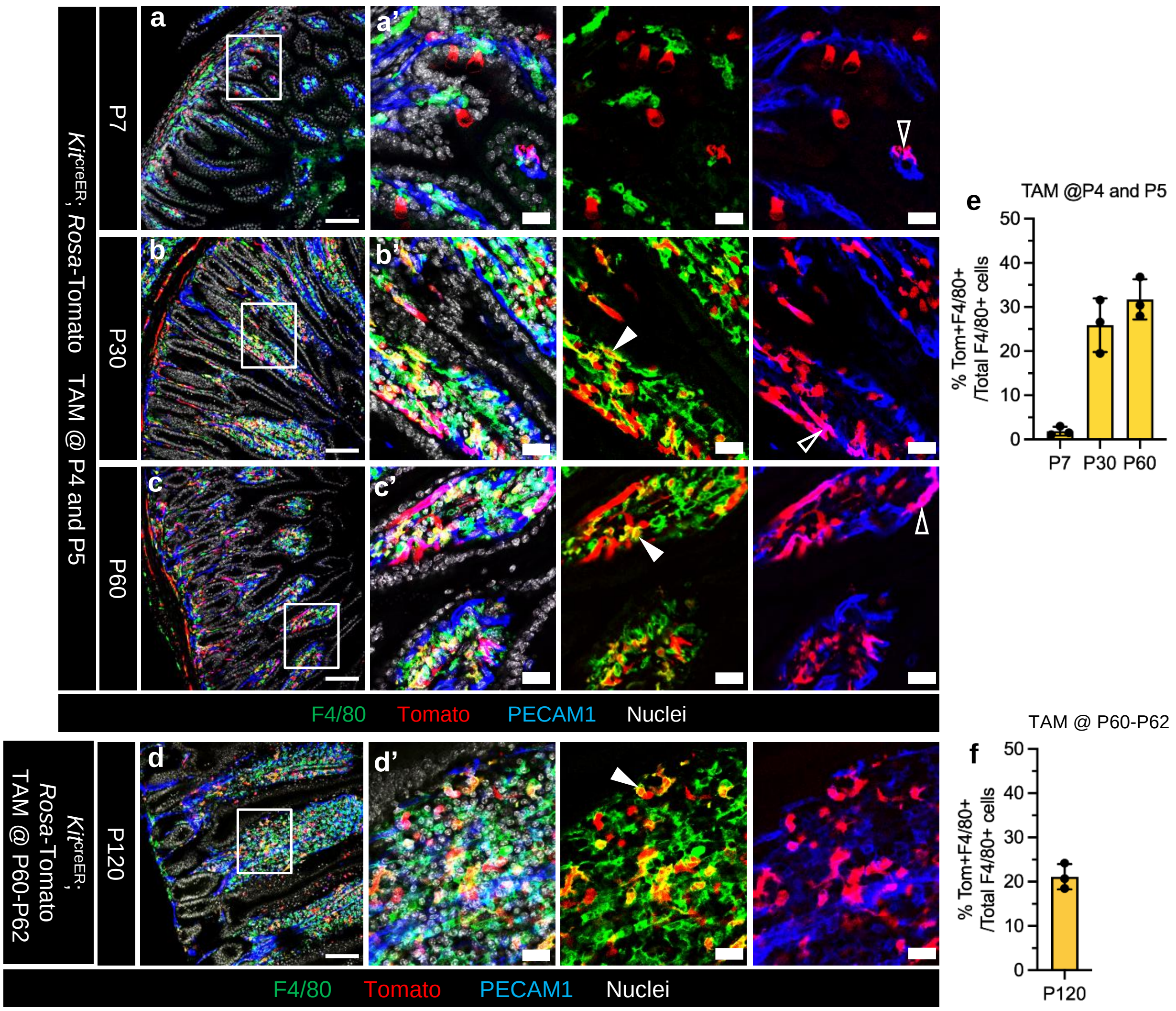

Supplementary Figure 8. Postnatal bone-marrow-derived HSCs contribute to juvenile and adult intestinal macrophages. Representative images (*n*=3 independent intestines) of intestine from P7 (a), P30 (b), P60 (c), and P120 (d) *Kit*^{creER}; *Rosa*-Tomato mice exposed to TAM at P4 and P5 (a-c) or P60-P62 (d) stained for markers of macrophages (F4/80) and endothelial cells (PECAM1). White arrowheads denote Tomato+ macrophages; black arrowheads denote Tomato+ endothelial cells. Thin scale bar, 100 µm; thick scale bar, 25 µm. (e) Graphs showing quantification (*n*=3) of percent Tomato-expressing F4/80+ macrophages in P7, P30, and P60 gut from *Kit*^{creER}; *Rosa*-Tomato mice exposed to TAM at P4 and P5. (f) Graph showing quantification (*n*=3) of percent Tomato-expressing F4/80+ macrophages in P120 gut from *Kit*^{creER}; *Rosa*-Tomato mice exposed to TAM at P60-P62. Data are shown as mean +/- SD.

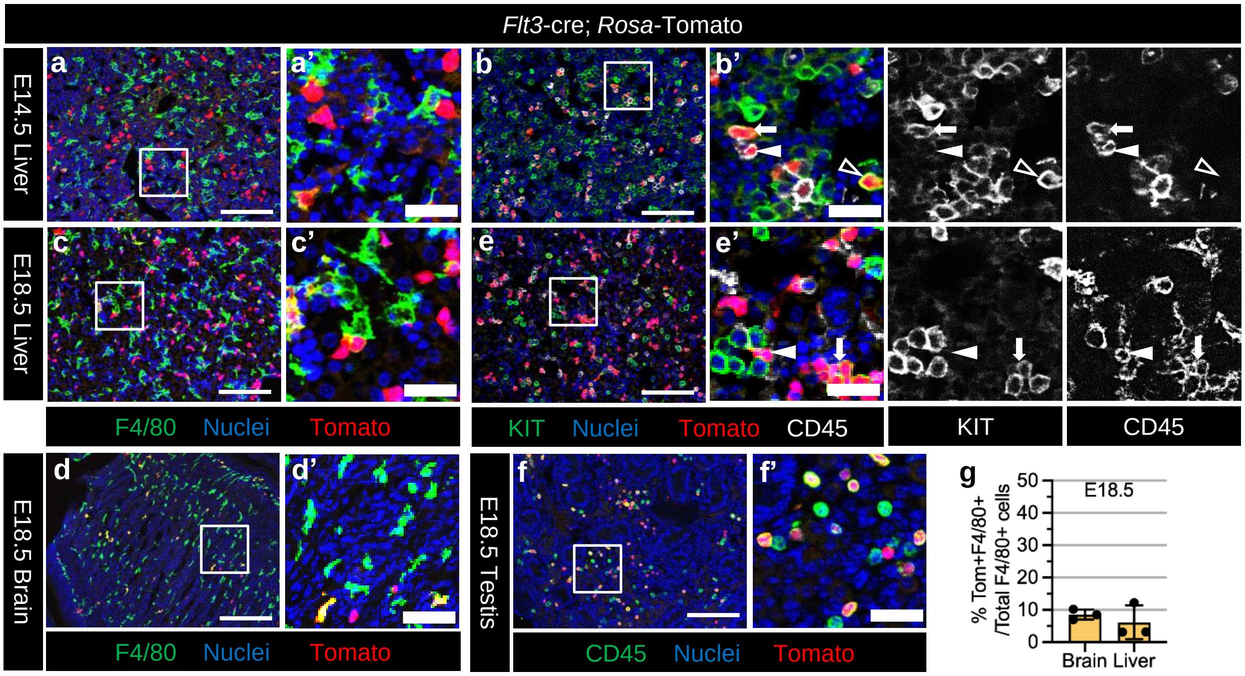

Supplementary Figure 9. *Flt3*-expressing fetal liver progenitors make a minor contribution to fetal brain microglia and liver Kupffer cells. (a-f) Representative images (*n*=3) of E14.5 liver (a, b), E18.5 liver (c, e), E18.5 brain (d) and E18.5 testis (f) from *Flt3*-cre; *Rosa*-Tomato embryos. Black arrowheads denote Tomato-expressing KIT+ CD45-negative cells; white arrowheads denote Tomato-expressing CD45+ KIT-negative cells; and white arrows denote Tomato-expressing CD45+ KIT+ cells. Thin scale bar, 100 µm; thick scale bar, 25 µm. (g) Graphs showing quantification (*n*=3) of percent Tomato-expressing F4/80+ macrophages in E18.5 brain or liver from *Flt3*-cre; *Rosa*-Tomato embryos. Data are shown as mean +/- SD.

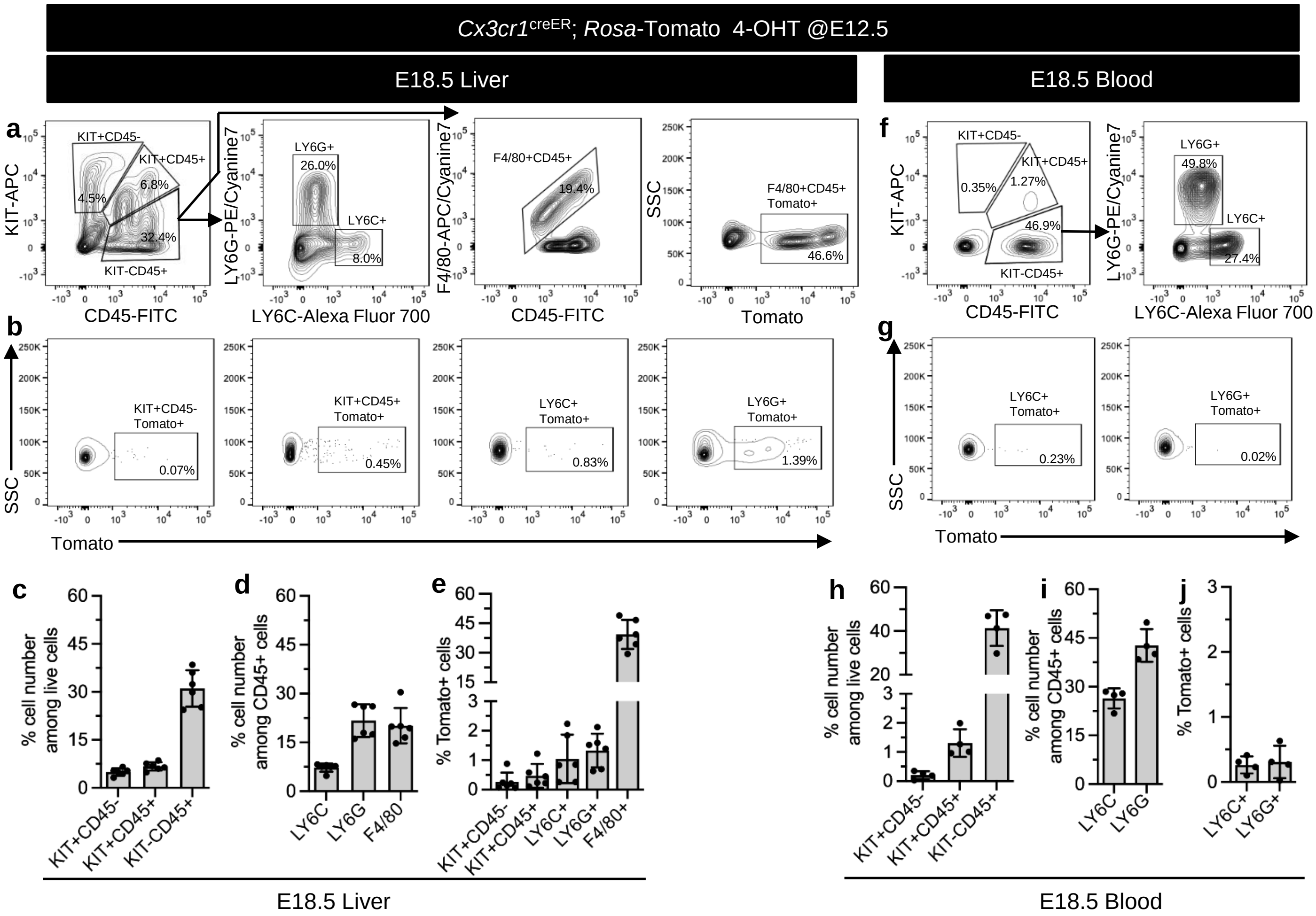

Supplementary Figure 10. *Cx3cr1*CreER-mediated lineage tracing shows minimal off-target labeling of HSCs, monocytes, and neutrophils in the fetal liver and blood. (a-j) Flow cytometric analyses of immune cell populations from fetal liver (a-e) and blood (f-j) of E18.5 *Cx3cr1*creER; *Rosa*-Tomato embryos exposed to 4-OHT at E12.5. (a) Isolation of fetal liver KIT+ cells and CD45+ cells into LY6G+ neutrophil, LY6C+ monocyte, F4/80+ macrophage, and F4/80+Tomato+ cell populations. (b) Tomato expression in fetal liver KIT+CD45-, KIT+CD45+, LY6C+, and LY6G+ cell populations. (c-e) Graphs showing percent cell number among live cells (c), percent cell number among CD45+ cells (d), and percent Tomato+ cells (e) of various fetal liver cell populations (*n*=6). (f) Isolation of blood KIT+ cells and CD45+ cells into LY6G+ neutrophil and LY6C+ monocyte populations. (g) Tomato expression in blood LY6C+ and LY6G+ cell populations. (h-j) Graphs showing percent cell number among live cells (h), percent cell number among CD45+ cells (i), and percent Tomato+ cells (j) of various blood cell populations (*n*=4). Data are shown as mean +/- SD.

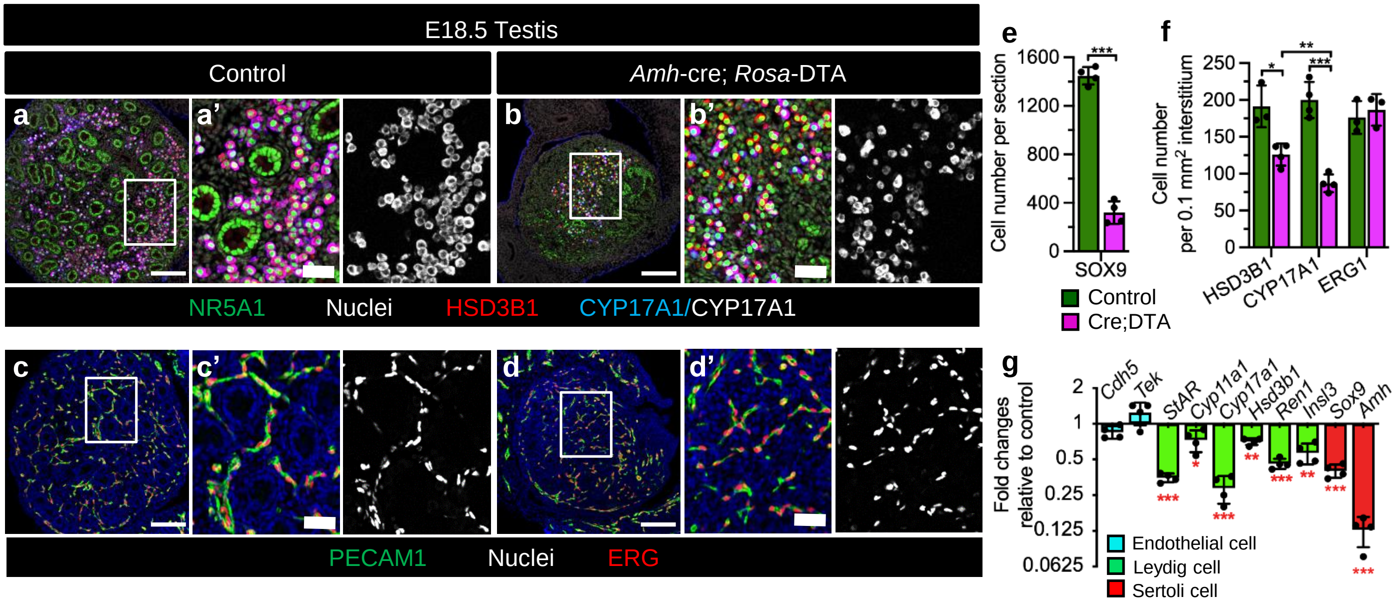

Supplementary Figure 11. Depletion of Sertoli cells by *Amh*-Cre;*Rosa*-DTA leads to a reduction in Leydig cells but has a negligible effect on endothelial cell number. (a-d) Images of E18.5 control (a, c) and *Amh*-cre; *Rosa*-DTA (b, d) fetal testes. NR5A1 labels Sertoli and Leydig cell nuclei; HSD3B1 and CYP17A1 label Leydig cells; and ERG labels endothelial cell nuclei. Thin scale bar, 100 µm; thick scale bar, 25 µm. Shown in (a, b) and (c, d) are representative images from *n*=4 and *n*=3 independent gonads, respectively. (e) Graph showing quantification (*n*=4 independent gonads) of SOX9+ (Sertoli) cells per gonadal optical section in E18.5 control versus *Amh*-cre; *Rosa*-DTA fetal testes. (f) Graphs showing quantification of HSD3B1+ (*n*=4 independent gonads), CYP17A1+ (*n*=4 independent gonads), or ERG1+ (*n*=3 independent gonads) cells per optical section per unit area of gonadal interstitium in E18.5 control versus *Amh*-cre; *Rosa*-DTA fetal testes. (g) qRT-PCR analyses (*n*=4 independent gonads) of whole E18.5 fetal testes showing fold change of expression in *Amh*-cre; *Rosa*-DTA testes versus controls for endothelial- (*Cdh5*, *Tek*), Leydig- (*StAR*, *Cyp11a1*, *Cyp17a1*, *Hsd3b1*, *Ren1*, *Insl3*), and Sertoli-specific (*Sox9*, *Amh*) genes. Data are shown as mean +/- SD. **P*<0.05; ***P*<0.01; ****P*<0.001 (two-tailed Student's *t*-test). Exact *P* values are provided in the Source Data file.

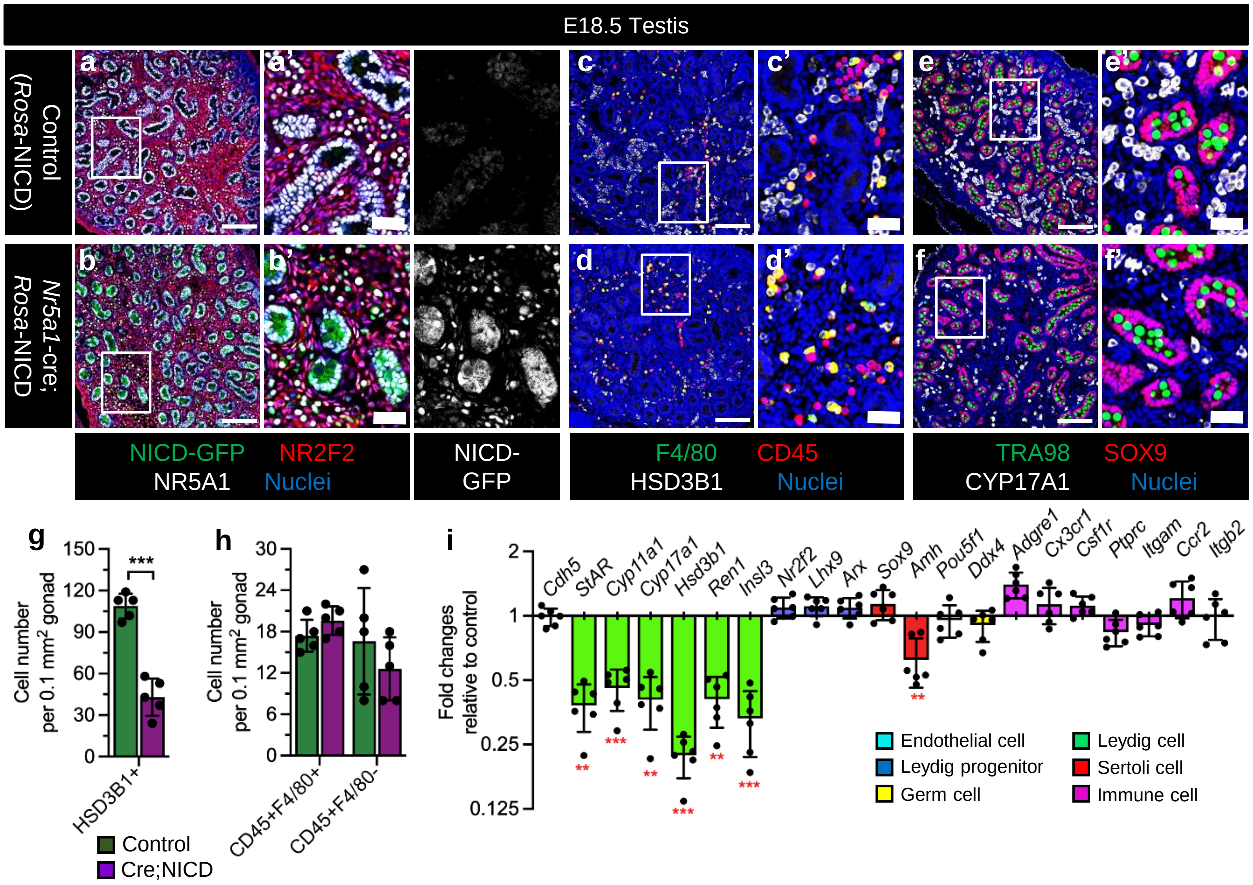

Supplementary Figure 12. Reduction of Leydig cells in *Nr5a1*-Cre;*Rosa*-NICD embryos does not impact immune cell numbers in the fetal testis. (a-f) Images of E18.5 control (a,c,e) and *Nr5a1*-cre; *Rosa*-NICD (b,d,f) fetal testes. Shown in (a, b) and (c-f) are representative images from *n*=3 and *n*=5 independent gonads, respectively. NICD-GFP labels cre-active, Notch-overexpressed testicular cells (from GFP cassette contained in *Rosa*-NICD construct); NR2F2 labels interstitial progenitor cells; and TRA98 labels germ cells. Thin scale bar, 100 µm; thick scale bar, 25 µm. (g) Graphs showing quantification (*n*=5 independent gonads) of HSD3B1+ Leydig cells per optical section per unit area of gonad in E18.5 control versus *Nr5a1*-cre; *Rosa*-NICD fetal testes. (h) Graphs showing quantification (*n*=5 independent gonads) of CD45+F4/80+ and CD45+F4/80- cells per optical section per unit area of gonad in E18.5 control versus *Nr5a1*-cre; *Rosa*-NICD fetal testes. (i) qRT-PCR analyses (*n*=6 independent gonads) of whole E18.5 fetal testes showing fold change of expression in *Nr5a1*-cre; *Rosa*-NICD testes versus controls for endothelial- (*Cdh5*), Leydig- (*StAR*, *Cyp11a1*, *Cyp17a1*, *Hsd3b1*, *Ren1*, *Insl3*), Leydig-progenitor- (*Nr2f2*, *Lhx9*, *Arx*), germ- (*Pou5f1*, *Ddx4*), Sertoli- (*Sox9*, *Amh*) and immune-specific (*Adgre1*, *Cx3cr1*, *Csf1r, Ptprc*, *Itgam*, *Ccr2*, *Itgb2*) genes. Data are shown as mean +/- SD. ***P*<0.01; ****P*<0.001 (two-tailed Student's *t*-test). Exact *P* values are provided in the Source Data file.

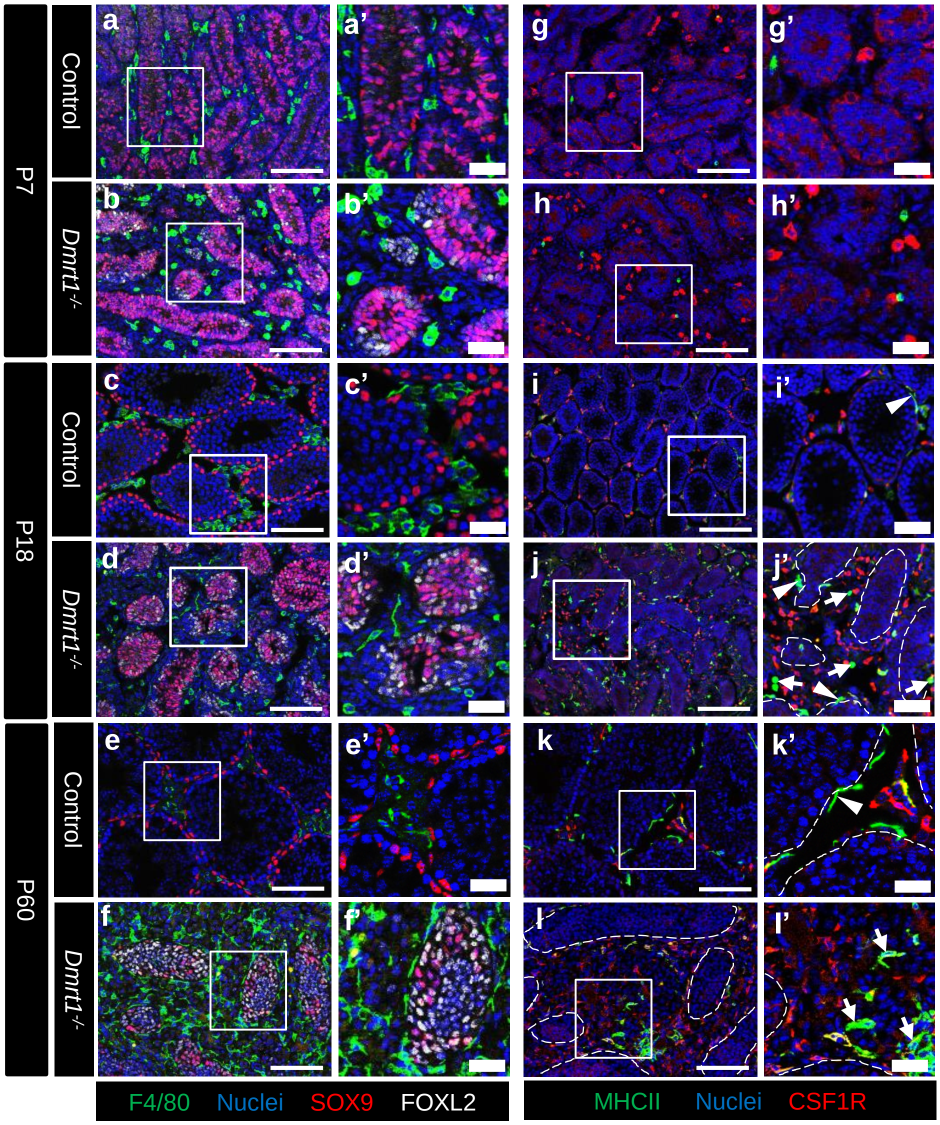

Supplementary Figure 13. Loss of Sertoli cell identity in *Dmrt1* KO testes inhibits peritubular macrophage differentiation and localization. (a-l) Representative images (*n*=3 independent testes) of P7 (a, b, g, h), P18 (c, d, i, j), and P60 (e, f, k, l) testes from XY *Dmrt1*$^{+/-}$ heterozygous control (a, c, e, g, i, k) and XY *Dmrt1*$^{-/-}$ KO (b, d, f, h, j, l) mice. SOX9 labels testicular Sertoli cells and FOXL2 labels ovarian granulosa cells. Dashed lines indicate seminiferous tubule boundaries. Arrowheads indicate MHCII+ peritubular macrophages with flattened morphology that are properly localized to the seminiferous tubule periphery; arrows indicate MHCII+ macrophages with improper interstitial localization and/or aberrant round morphology. Thin scale bar, 100 µm; thick scale bar, 25 µm.

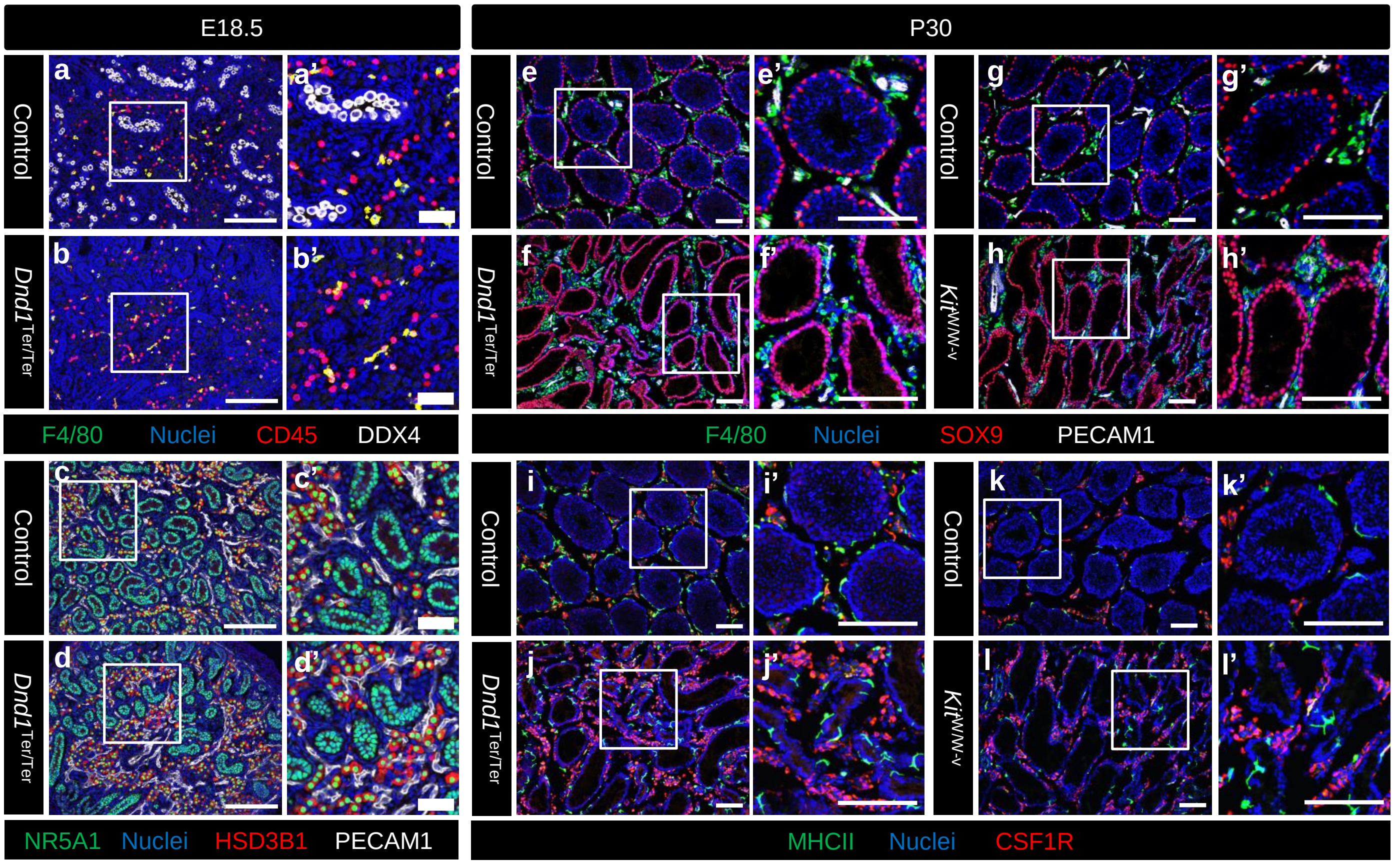

Supplementary Figure 14. Germ cells are not required for fetal testicular monocyte recruitment or macrophage differentiation. (a-d) Representative images (*n*=3 independent testes) of E18.5 control (a, c) and *Dnd1*$^{Ter/Ter}$ (b, d) fetal testes. (e-l) Representative images (*n*=3 independent testes) of P30 control (e, g, i, k), *Dnd1*$^{Ter/Ter}$ mutant (f, j), and *Kit*$^{W/W\text{-}v}$ mutant (h, l) juvenile testes. Thin scale bar, 100 µm; thick scale bar, 25 µm.

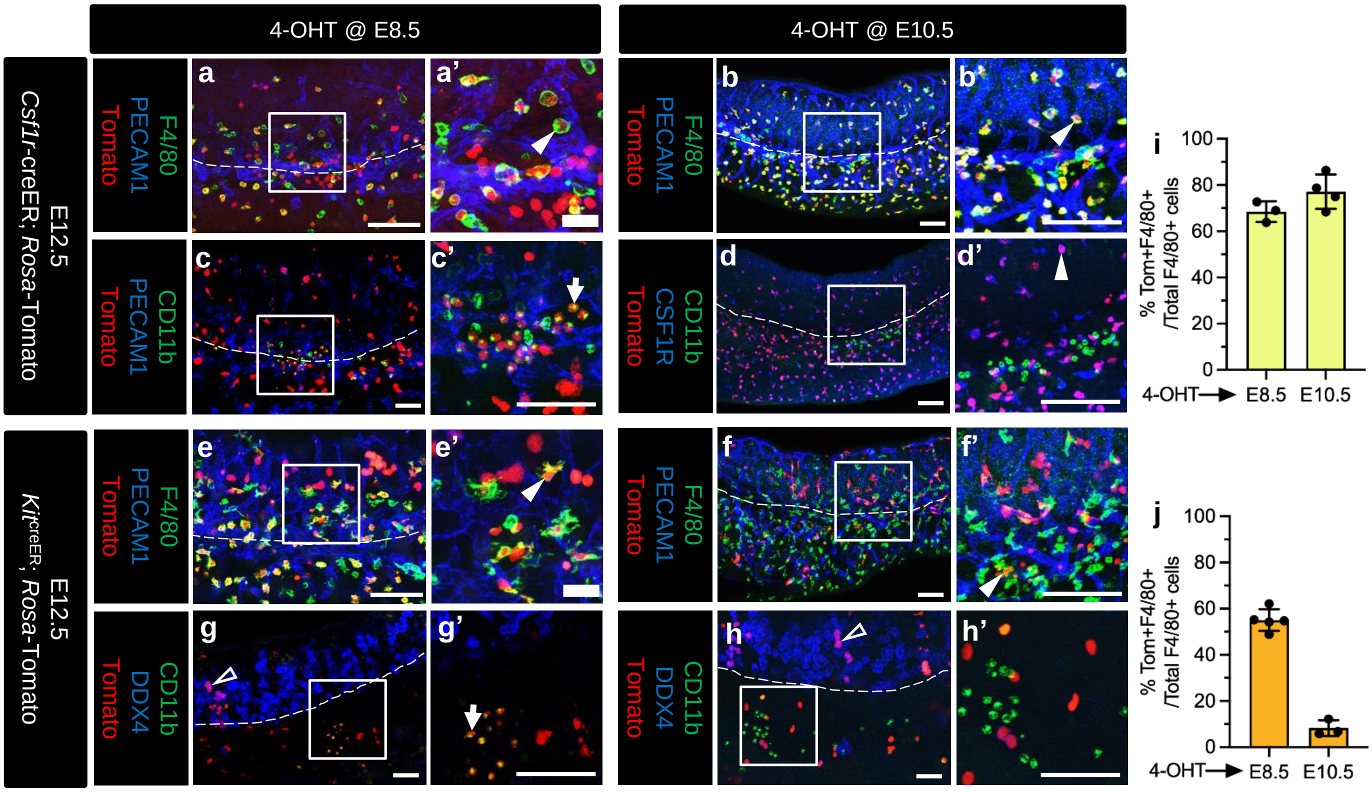

Supplementary Figure 15. YS-derived EMPs give rise to early gonadal monocytes and macrophages. (a-h) Representative images (*n*=3 independent testes) of E12.5 fetal testes from *Csf1r*-creER; *Rosa*-Tomato (a-d) and *Kit*^{creER}; *Rosa*-Tomato (e-h) embryos exposed to 4-OHT at E8.5 (a, c, e, g) or E10.5 (b, d, f, h) stained for various markers such as F4/80 (macrophages), CD11b (myeloid cells such as monocytes and granulocytes), CSF1R (macrophages), PECAM1 (germ cells and endothelial cells), and DDX4 (germ cells). White arrowheads indicate Tomato-expressing F4/80+ or CSF1R+ macrophages; black arrowheads indicate Tomato-expressing DDX4+ germ cells; and white arrows indicate Tomato-expressing CD11b+ cells (likely monocytes). Thin scale bar, 100 µm; thick scale bar, 25 µm. (i, j) Graphs showing quantification of percent Tomato-expressing F4/80+ macrophages in E12.5 *Csf1r*-creER; *Rosa*-Tomato (i) fetal testes exposed to 4-OHT at E8.5 (*n*=3 independent testes) or E10.5 (*n*=4 independent testes) and *Kit*^{creER}; *Rosa*-Tomato (j) fetal testes exposed to 4-OHT at E8.5 (*n*=4 independent testes) or E10.5 (*n*=3 independent testes). Data are shown as mean +/- SD.

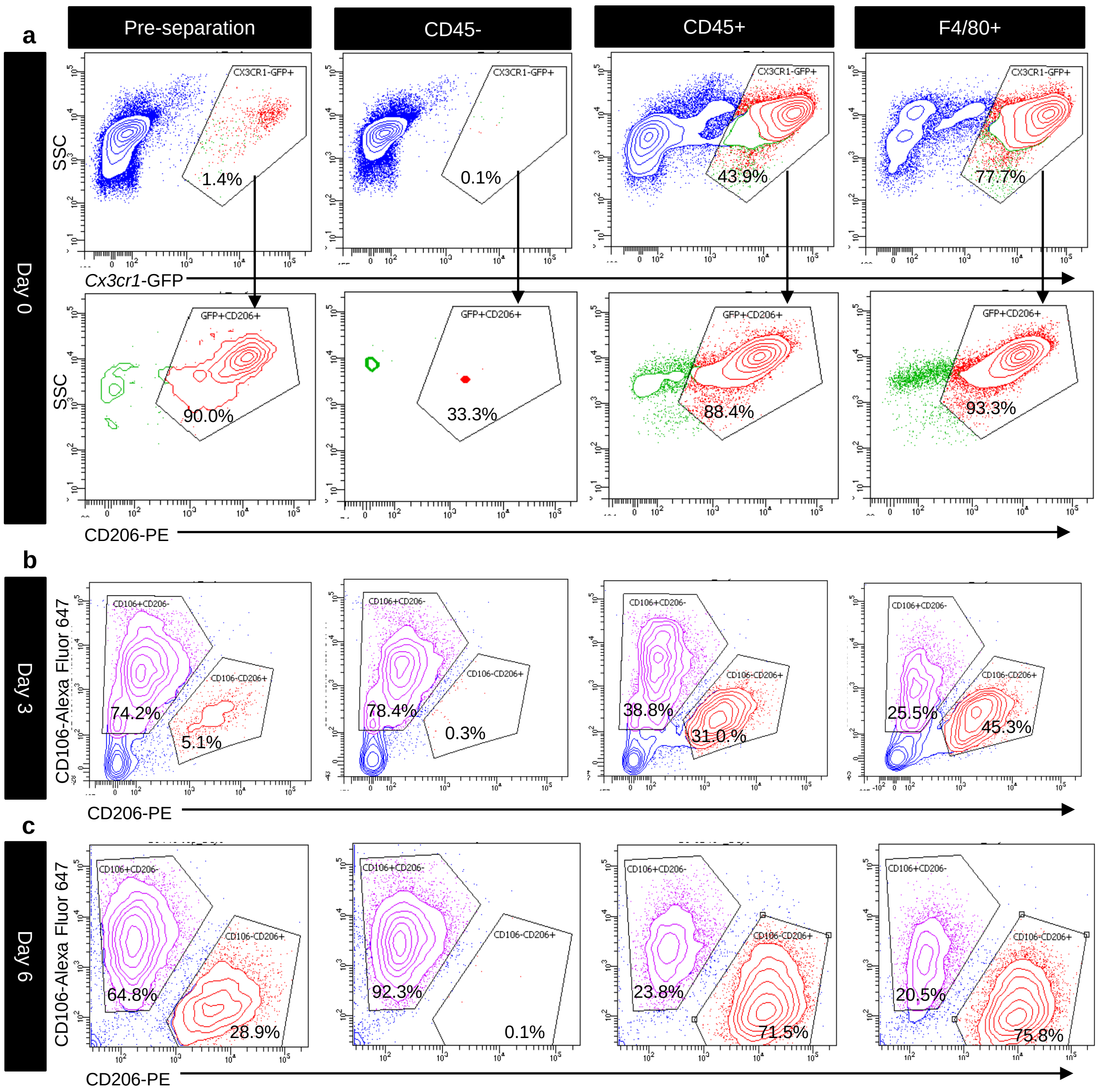

Supplementary Figure 16. Flow cytometric analyses reveal purity and cellular composition of cell populations used for in vitro culture assays. (a-c) Flow cytometry plots for 4 different cell populations (pre-separation, CD45-depleted, CD45-enriched, and F4/80-enriched) isolated from *Cx3cr1*$^{GFP/+}$ adult testes at day 0 (a) or C57BL/6J adult testes at day 3 (b) and day 6 (c) of in vitro culture. Cells were analyzed for markers of macrophages (GFP from *Cx3cr1*^{GFP}), adult interstitial macrophages (CD206), and adult Leydig cells (CD106, also known as VCAM1). SSC denotes side scatter.

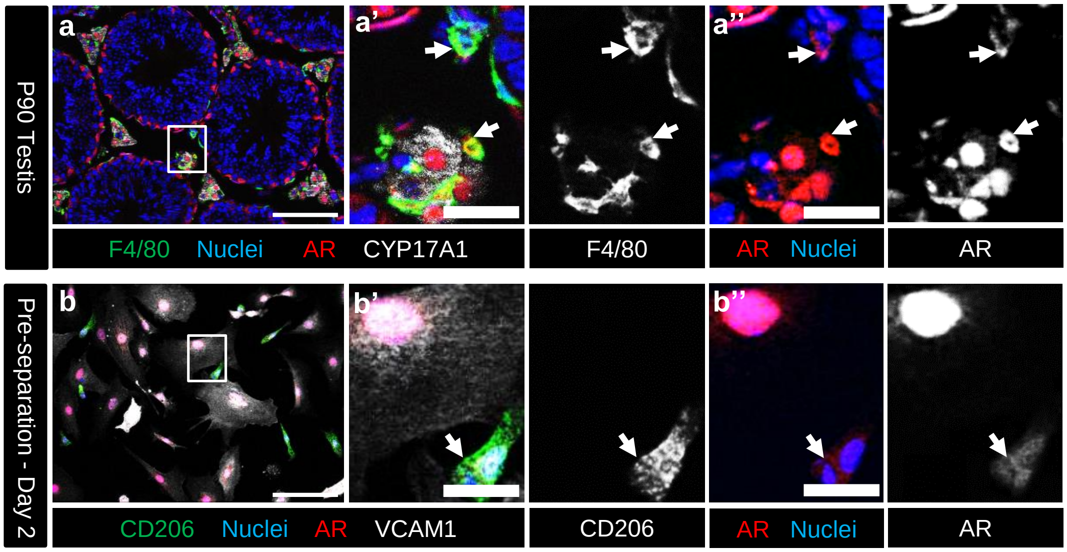

Supplementary Figure 17. Androgen receptor (AR) is expressed in the cell membrane of adult interstitial macrophages. Representative images of P90 adult C57BL/6J intact testis (a) (*n*=3 independent testes) and pre-separation population of testicular cells isolated from adult C57BL/6J testis after 2 days of in vitro culture (b) (*n*=3 independent experiments). Arrows indicate F4/80+ or CD206+ interstitial macrophages that express AR. In contrast to Leydig cells (VCAM1+ or CYP17A1+), which express AR predominantly in the nucleus, macrophage expression of AR is localized more broadly throughout the cell and in the cell membrane. Thin scale bar, 100 µm; thick scale bar, 25 µm.

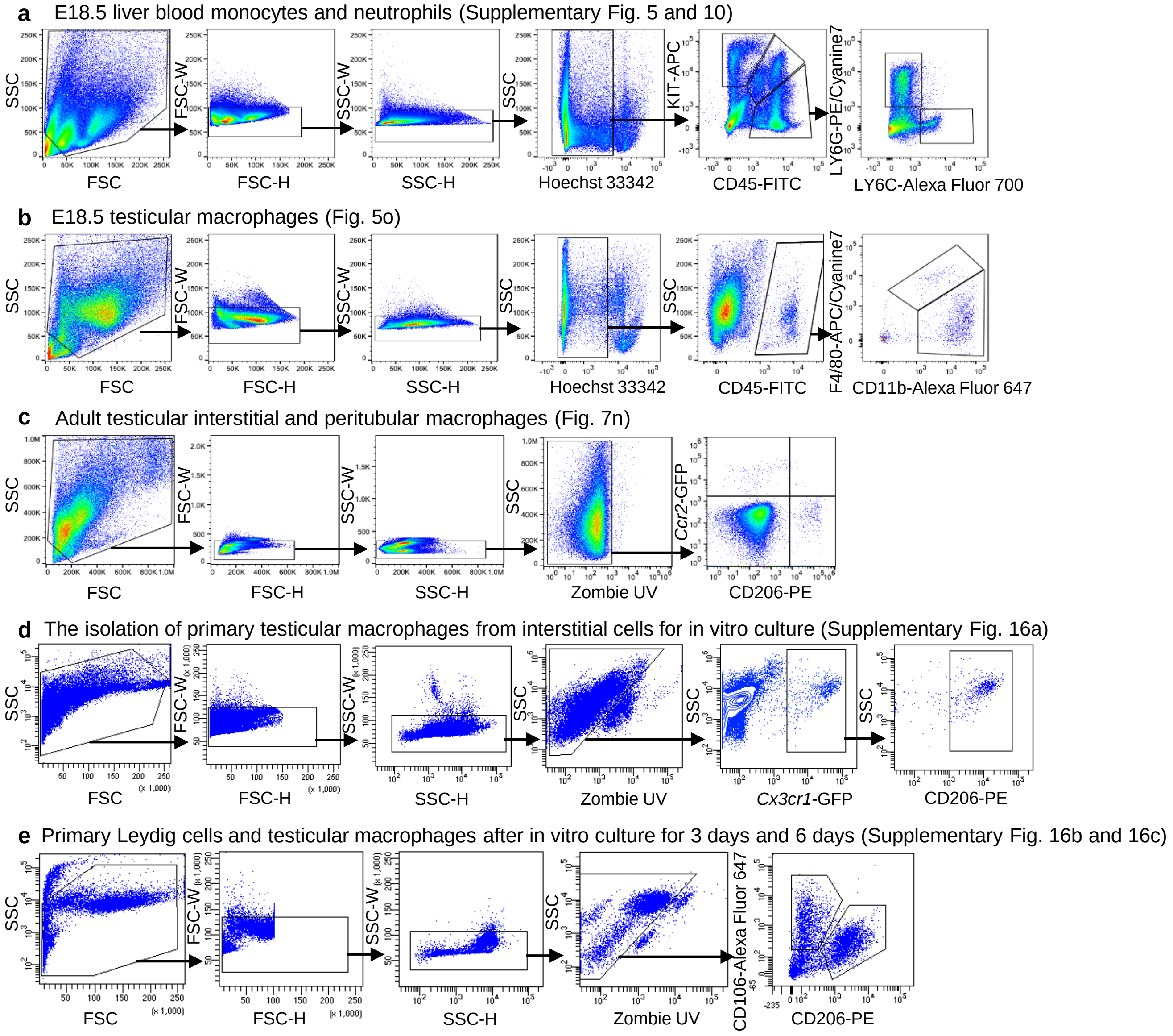

Supplementary Figure 18. Gating strategies used for flow cytometric analyses. (a-e) Representative flow cytometry plots demonstrating basic gating strategies used in various experiments in this study. (a) Kit+ HSCs and different leukocyte populations in E18.5 fetal liver and blood. (b) F4/80-hi CD11b-hi macrophages and F4/80-lo CD11b-hi monocytes in E18.5 fetal testis. (c) CD206+GFP- testicular interstitial macrophages and CD206-GFP+ testicular peritubular macrophages in adult *Ccr2*$^{GFP/+}$ mice. (d) CD206+GFP+ testicular macrophages in adult *Cx3cr1*$^{GFP/+}$ mice. (e) CD106+CD206- Leydig cells and CD106-CD206+ testicular macrophages in in vitro culture of interstitial cells for 3 days or 6 days. All cells are initially gated for single cells (FSC-W vs FSC-H and SSC-W vs SSC-H) and live cells (Hoechst-33342-negative or Zombie-UV-negative) before downstream analyses.

SUPPLEMENTARY TABLES

Supplementary Table 1. Primary antibodies for immunofluorescence (IF) and flow cytometry (FC).

Primary Antibody (IF)	Dilution	Source/Reference
Goat anti-AMH	1:500	Santa Cruz #sc-6886
Rabbit anti-AR	1:300	Santa Cruz #sc-816
Rat anti-B220	1:400	eBioscience #14–0452-81
Rat anti-CD11b	1:250	BD Pharmingen #557395
Rat anti-CD206	1:1,000	AbD Serotec #MCA2235T
Rat anti-CD4	1:400	BioLegend #100505
Goat anti-CD45	1:500	R&D #AF114
Rat anti-CD45	1:300	BioLegend #103101
Rabbit anti-Cleaved Caspase 3 (Asp175)	1:250	Cell Signaling #9661S
Rabbit anti-CSF1R	1:500	Santa Cruz #sc-692
Rabbit anti-CYP11A1	1:500	D. Wilhelm[1]
Goat anti-CYP17A1	1:500	Santa Cruz #sc-46081
Rabbit anti-DDX4	1:1,000	Abcam #ab13840
Rabbit anti-ERG	1:250	Abcam #ab92513
Rat anti-F4/80	1:2,000	AbD Serotec #MCA497RT
Goat anti-FOXL2	1:250	Novus #100-1277
Chicken anti-GFP	1:1,000	Aves #GFP-1020
Rat anti-GR1	1:500	AbD Serotec #MCA2387T
Rabbit anti-HSD3B1	1:500	Cosmo Bio #KAL-KO607
Rabbit anti-IBA1	1:1,000	Wako #019–19741
Goat anti-KIT	1:400	R&D #AF1356
Rat anti-MHCII	1:500	eBioscience #14–5321-81
Rat anti-MKI67	1:500	ThermoFisher #14-5698-80
Mouse anti-NR2F2	1:500	R&D #PP-H7147-00
Rat anti-NR5A1 (SF1)	1:250	Cosmo Bio #KAL-KO610
Goat anti-PECAM1	1:250	R&D #AF3628
Rat anti-PECAM1	1:250	BD Pharmingen #553370
Rabbit anti-SOX9	1:3,000	Millipore #AB5535
Rat anti-TRA98	1:1,000	Abcam #ab82527
Goat anti-VCAM1	1:2,000	R&D #AF643

Primary Antibody (FC)	Dilution	Source/Reference
Alexa Fluor 647 anti-mouse CD106 (VCAM1)	1:100	BioLegend #105712
APC anti-mouse CD117 (KIT)	1:100	BioLegend #105811
Alexa Fluor 647 anti-mouse CD11b	1:100	BioLegend #101220
PE anti-mouse CD206	1:200	BioLegend #141705
FITC anti-mouse CD45	1:100	BioLegend #103107
APC/Cyanine7 anti-mouse F4/80	1:100	BioLegend #123117
Alexa Fluor 700 anti-mouse Ly-6C	1:100	BioLegend #128023
PE/Cyanine7 anti-mouse Ly-6G	1:100	Biolegend #127617

Supplementary Table 2. Sequences of primers used for qRT-PCR analyses.

Gene name	Sequence (5’ to 3’)
Adgre1 (F4/80) forward	CCCCAGTGTCCTTACAGAGTG
Adgre1 (F4/80) reverse	GTGCCCAGAGTGGATGTCT
Amh forward	CCACACCTCTCTCCACTGGTA
Amh reverse	GGCACAAAGGTTCAGGGGG
Arx forward	CAAGGATGGTGAGGACAGC
Arx reverse	TCTGGAACCACACCTGGACT
Ccr2 forward	ACACCCTGTTTCGCTGTAGG
Ccr2 reverse	TGGCCTGGTCTAAGTGCTTG
Cdh5 forward	TCCTCTGCATCCTCACTATCACA
Cdh5 reverse	GTAAGTGACCAACTGCTCGTGAAT
Csf1r forward	TGTCATCGAGCCTAGTGGC
Csf1r reverse	CGGGAGATTCAGGGTCCAAG
Cx3cr1 forward	GAGTATGACGATTCTGCTGAGG
Cx3cr1 reverse	CAGACCGAACGTGAAGACGAG
Cyp11a1 forward	TGGCCCCATTTACAGGGAGAA
Cyp11a1 reverse	GGCATCTGAACTCTTAAACAGGA
Cyp17a1 forward	CAGAGAAGTGCTCGTGAAGAAG
Cyp17a1 reverse	AGGAGCTACTACTATCCGCAAA
Ddx4 forward	TACTGTCAGACGCTCAACAGGA
Ddx4 reverse	ATTCAACGTGTGCTTGCCCT
Gapdh forward	AGGTCGGTGTGAACGGATTTG
Gapdh reverse	TGTAGACCATGTAGTTGAGGTCA
Hsd3b1 forward	CAAGTGTGCCAGCCTTCATCT
Hsd3b1 reverse	TTCATGATTCTGTTCCTCGTGG
Itgam forward	CCACACTAGCATCAAGGGCA
Itgam reverse	AAGGGACACACTGACACCTG
Itgb2 forward	GTGTCCCAGGAATGCACCAA
Itgb2 reverse	TATCATCGGCTGGACAACCC
Insl3 forward	CCTCCTGGCTATGTCATTGC
Insl3 reverse	CCTGTGGTCCTTGCTTACTG
Kit forward	CATGGCGTTCCTCGCCT
Kit reverse	GCCCGAAATCGCAAATCTTT
Lhx9 forward	TGTAATGCCCCAAGATTTGTTCTCCC
Lhx9 reverse	ACCAGCAGCCTTATCCACCTTCACAG
Nr2f2 forward	TCAACTGCCACTCGTACCTG
Nr2f2 reverse	CCATGATGTTGTTAGGCTGCA
Pou5f1 forward	GGAGGAAGCCGACAACAATGA
Pou5f1 reverse	TCCACCTCACACGGTTCTCAA
Ptprc forward	GGAGGACACAGCACATTGGA
Ptprc reverse	CCCCTGAGCAGCAATCATCA
Ren1 forward	TGCTGGCCAAGTTTGACGGTG
Ren1 reverse	CACCTCGCCCCCCAGCAG
Sox9 forward	GCGGAGCTCAGCAAGACTCTG
Sox9 reverse	ATCGGGGTGGTCTTTCTTGTG
StAR forward	TACATCCAGCAGGGAGAGGTG
StAR reverse	CAGCGCACGCTCACGAAGTCT

Tek forward	CGGCCAGGTACATAGGAGGAA
Tek reverse	TCACATCTCCGAACAATCAGC

SUPPLEMENTARY REFERENCES

1 Svingen, T., Francois, M., Wilhelm, D. & Koopman, P. Three-dimensional imaging of Prox1-EGFP transgenic mouse gonads reveals divergent modes of lymphangiogenesis in the testis and ovary. *PLoS One* **7**, e52620, doi:10.1371/journal.pone.0052620 (2012).
