# Supplementary material for: Testicular macrophages are recruited during a narrow fetal time window and promote organ-specific developmental functions
Source: Nat Commun. 2023 Mar 15;14:1439. doi: 10.1038/s41467-023-37199-0 (PMC10017703; doi:10.1038/s41467-023-37199-0)
Supplement: Supplementary file 3 — Reporting Summary [file 41467_2023_37199_MOESM3_ESM.pdf]

nature portfolio

Corresponding author(s): Tony DeFalco

Last updated by author(s): Feb 14, 2023

Reporting Summary

Nature Portfolio wishes to improve the reproducibility of the work that we publish. This form provides structure for consistency and transparency in reporting. For further information on Nature Portfolio policies, see our Editorial Policies and the Editorial Policy Checklist.

Statistics

For all statistical analyses, confirm that the following items are present in the figure legend, table legend, main text, or Methods section.

n/a	Confirmed	
☐	☒	The exact sample size (*n*) for each experimental group/condition, given as a discrete number and unit of measurement
☐	☒	A statement on whether measurements were taken from distinct samples or whether the same sample was measured repeatedly
☐	☒	The statistical test(s) used AND whether they are one- or two-sided<br>*Only common tests should be described solely by name; describe more complex techniques in the Methods section.*
☒	☐	A description of all covariates tested
☒	☐	A description of any assumptions or corrections, such as tests of normality and adjustment for multiple comparisons
☐	☒	A full description of the statistical parameters including central tendency (e.g. means) or other basic estimates (e.g. regression coefficient) AND variation (e.g. standard deviation) or associated estimates of uncertainty (e.g. confidence intervals)
☐	☒	For null hypothesis testing, the test statistic (e.g. *F*, *t*, *r*) with confidence intervals, effect sizes, degrees of freedom and *P* value noted<br>*Give P values as exact values whenever suitable.*
☒	☐	For Bayesian analysis, information on the choice of priors and Markov chain Monte Carlo settings
☒	☐	For hierarchical and complex designs, identification of the appropriate level for tests and full reporting of outcomes
☒	☐	Estimates of effect sizes (e.g. Cohen's *d*, Pearson's *r*), indicating how they were calculated

Our web collection on statistics for biologists contains articles on many of the points above.

Software and code

Policy information about availability of computer code

Data collection	All immunofluorescence images were obtained using Volocity (PerkinElmer, version 6.3) or Nikon's NIS-Elements AR (Advanced Research) software. All quantification data were obtained using Image J software (32-bit Java 1.8.0_241); flow cytometric data were obtained using FACS Diva (BD Biosciences; version 9.0) and FlowJo (BD Biosciences; version 10.8) software; and qRT-PCR data were collected by using StepOnePlus software (Thermo Fisher; version 2.3).
Data analysis	All statistical analyses for quantification, qRT-PCR, flow cytometry and testosterone concentration data were performed in Microsoft Excel 2013 (v15.0) and GraphPad Prism (version 8.0).

For manuscripts utilizing custom algorithms or software that are central to the research but not yet described in published literature, software must be made available to editors and reviewers. We strongly encourage code deposition in a community repository (e.g. GitHub). See the Nature Portfolio guidelines for submitting code & software for further information.

Data

Policy information about availability of data

All manuscripts must include a data availability statement. This statement should provide the following information, where applicable:

- Accession codes, unique identifiers, or web links for publicly available datasets
- A description of any restrictions on data availability
- For clinical datasets or third party data, please ensure that the statement adheres to our policy

Source data for quantitative assays (e.g., graphs) in Figures 1-7 and Supplementary Figures 1-3, 5, 6, 8-12, and 15 are provided with the paper in the Source Data

file. Other data supporting the findings of this study are available within the paper and its supplementary information files, and they are also available from the corresponding author upon request.

Human research participants

Policy information about studies involving human research participants and Sex and Gender in Research.

Reporting on sex and gender	N/A. No human research participants were included in this study.
Population characteristics	N/A
Recruitment	N/A
Ethics oversight	N/A

Note that full information on the approval of the study protocol must also be provided in the manuscript.

Field-specific reporting

Please select the one below that is the best fit for your research. If you are not sure, read the appropriate sections before making your selection.

[x] Life sciences [ ] Behavioural & social sciences [ ] Ecological, evolutionary & environmental sciences

For a reference copy of the document with all sections, see nature.com/documents/nr-reporting-summary-flat.pdf

Life sciences study design

All studies must disclose on these points even when the disclosure is negative.

Sample size	No power analyses were performed to determine sample size. At least 3 (usually 3-6) independent biological replicates were used for all experiments. The number of biological replicates were determined based on the minimum number required to perform statistical analyses.
Data exclusions	No data were excluded.
Replication	All experiments were replicated successfully. Immunofluorescence experiments were performed on at least 3 independent, individual animals, and at least 3 tissue sections of each biological replicate were used to count cell numbers of different cell types at each time point. Flow cytometric analyses for E18.5 tissues were performed on least 3 independent embryos from 2-3 litters. In qRT-PCR experiments, at least two gonads from independent embryos were pooled for each biological replicate. For primary cell culture in vitro, each cell population was cultured in 2-3 wells for each biological replicate. For testis cord morphometric analyses, 9-11 whole fetal gonads from independent embryos from at least 3 independent litters were used, whereby five testis cords of each whole mount gonad were measured.
Randomization	Samples were allocated into experimental groups by genotype or treatment condition.
Blinding	No specific methods were used for blinding in this study, although virtually all experiments were performed by multiple authors to virtually identical results.

Reporting for specific materials, systems and methods

We require information from authors about some types of materials, experimental systems and methods used in many studies. Here, indicate whether each material, system or method listed is relevant to your study. If you are not sure if a list item applies to your research, read the appropriate section before selecting a response.

Materials & experimental systems

n/a	Involved in the study	
[ ]	[x]	Antibodies
[x]	[ ]	Eukaryotic cell lines
[x]	[ ]	Palaeontology and archaeology
[ ]	[x]	Animals and other organisms
[x]	[ ]	Clinical data
[x]	[ ]	Dual use research of concern

Methods

n/a	Involved in the study	
[x]	[ ]	ChIP-seq
[ ]	[x]	Flow cytometry
[x]	[ ]	MRI-based neuroimaging

Antibodies

Antibodies used	The following antibodies were used for immunofluorescence: Rabbit anti-CYP11A1 (1:500, a gift from Dr. Dagmar Wilhelm at The University of Melbourne, reported in Svingen et al., 2012; PMID: 23285114); Rat anti-F4/80 (1:2,000, AbD Serotec #MCA497RT); Rabbit anti-IBA1 (1:1,000, Wako #019–19741); Goat anti-FOXL2 (1:250, Novus #100-1277); Rabbit anti-CSF1R (1:500, Santa Cruz #sc-692); Rat anti-MHCII (1:500, eBioscience #14–5321-81); Rat anti-CD45 (1:300, BioLegend #103101); Goat anti-CD45 (1:500, R&D #CD45); Rat anti-CD4 (1:400, BioLegend #100505); Rat anti-B220 (1:400, eBioscience #14–0452-81); Rat anti-GR1 (1:500, AbD Serotec #MCA2387T); Rat anti-NR5A1 (1:250, Cosmo Bio #KAL-KO610); Goat anti-PECAM1 (1:250, R&D #AF3628); Rat anti-PECAM1 (1:250, BD Pharmingen #553370); Goat anti-KIT (1:400, R&D #AF1356); Goat anti-AMH (1:500, Santa Cruz #sc-6886); Rabbit anti-SOX9 (1:3,000, Millipore #AB5535); Rat anti-CD206 (1:1,000, AbD Serotec #MCA2235T); Goat anti-VCAM1 (1:2,000, R&D #AF643); Rat anti-CD11b (1:250, BD Pharmingen #557395); Rabbit anti-DDX4 (1:1,000, Abcam #ab13840); Goat anti-CYP17A1 (1:500, Santa Cruz #sc-46081); Rat anti-TRA98 (1:1,000, Abcam #ab82527); Rabbit anti-AR (1:300, Santa Cruz #sc-816); Rabbit anti-ERG (1:500, Abcam #ab92513); Mouse anti-NR2F2 (1:500, R&D #PP-H7147-00); Rabbit anti-HSD3B1 (1:500, Cosmo Bio #KAL-KO607); Rabbit anti-Cleaved Caspase 3 (1:250, Cell Signaling #9661S); Rat anti-MKI67 (1:1,000, ThermoFisher #14-5698-80); and Chicken anti-GFP (1:1,000, Aves #GFP-1020). The following antibodies were used for flow cytometry: Alexa Fluor® 647 anti-mouse VCAM1/CD106 (1:100, Biolegend #105712); PE anti-mouse CD206 (1:200, Biolegend #141705); APC anti-mouse CD117 (1:100, Biolegend #105811); FITC anti-mouse CD45 (1:100, Biolegend #103107); Alexa Fluor® 700 anti-mouse Ly-6C (1:100, Biolegend #128023); PE/Cyanine7 anti-mouse Ly-6G (1:100, Biolegend #127617); APC/Cyanine7 anti-mouse F4/80 (1:100, Biolegend #123117); and Alexa Fluor® 647 anti-mouse CD11b (1:100, Biolegend #101220).
Validation	Commercially available antibodies have provided validation statements on Research Resource Identification (RRID) Portal or their respective websites.<br><br>F4/80, Bio-Rad Cat# MCA497RT, RRID:AB_1102558, Clone ID: CI:A3-1,Host Organism: rat, Clonality monoclonal, Application: WB, ChIP, IF, IHC-Fr, IHC-P<br><br>IBA1, FUJIFILM Wako Shibayagi Cat# 019-19741, RRID:AB_839504, Host : rabbit, Clonality polyclonal, Application: ICC, IHC-Fr<br><br>FOXL2, Novus Cat# NB100-1277, RRID:AB_2106188, Host: goat, Clonality polyclonal, Application: WB, ChIP, ICC/IF, IHC, IHC-Fr, IHC-P<br><br>c-Fms/CSF-1R (C-20), Santa Cruz Biotechnology Cat# sc-692, RRID:AB_631025, Discontinued: 2016, Host: rabbit, Clonality polyclonal, Application: WB, IP, IF, ELISA<br><br>MHC Class II (I-A/I-E), Thermo Fisher Scientific Cat# 14-5321-81, RRID:AB_467560, Clone ID: M5/114.15.2, Host: rat, Clonality monoclonal, Application: IF, IHC-Fr, IHC-P,WB, FC<br><br>CD45, BioLegend Cat# 103101, RRID:AB_312966, Clone ID: 30-F11, Host : Rat, Clonality monoclonal, Application: FC, IHC-F, IP, IHC, WB<br><br>CD45, R and D Systems Cat# AF114, RRID:AB_442146, Host : goat, Clonality polyclonal, Application: FC, IHC, WB<br><br>CD4, BioLegend Cat# 100505, RRID:AB_312708, Clone ID: RM4-5, Host : Rat, Clonality monoclonal, Application: FC, IHC-F, IHC<br><br>CD45R (B220), Thermo Fisher Scientific Cat# 14-0452-81, RRID:AB_467253, Clone ID: RA3-6B2, Host: rat, Clonality monoclonal, Application: IHC (P), IP, Flow, IHC (F)<br><br>GR1, Bio-Rad Cat# MCA2387T, RRID:AB_2115659, Clone ID: RB6-8C5, Host : rat, Clonality monoclonal, Application: IIHC-F; FC; WB; IP<br><br>NR5A1 (SF1), Cosmo Bio #KAL-KO610, Clone ID: 1B1F10, Host: Rat, Clonality monoclonal, Application: ICC, IF, IHC, WB<br><br>PECAM-1 (CD31), R and D Systems Cat# AF3628, RRID:AB_2161028, Host: goat, Clonality polyclonal, Application: WB, FC, IIHC<br><br>PECAM-1, BD Biosciences Cat# 553370, RRID:AB_394816, Clone ID: MEC 13.3, Host: rat, Clonality monoclonal, Application: FC, IHC-F, IF<br><br>SCF-R/c-KIT, R and D Systems Cat# AF1356, RRID:AB_354750, Host: goat, Clonality polyclonal, Application: WB, FC, IHC<br><br>AMH/MIS (C-20), Santa Cruz Biotechnology Cat# sc-6886, RRID:AB_649207, Discontinued: 2016, Host: goat, Clonality polyclonal, Application: WB, IP, IF, IHC(P)<br><br>SOX9, Millipore Cat# AB5535, RRID:AB_2239761, Host: Rabbit, Clonality polyclonal, Application: IHC, WB, ChIP, ICC, IF<br><br>CD206, Bio-Rad Cat# MCA2235T, RRID:AB_1101333, Clone ID: MR5D3, Host Organism: rat, Clonality monoclonal, Application: FC, IHC-F, IP, WB<br><br>VCAM-1/CD106, R and D Systems Cat# AF643, RRID:AB_355499, Host Organism: goat, Clonality polyclonal, Application: WB, IHC<br><br>CD11b, BD Biosciences Cat# 557395, RRID:AB_2296385, Clone ID: M1/70, Host Organism: rat, Clonality monoclonal, Application: FC, IF, WB

DDX4/MVH, Abcam Cat# ab13840, RRID:AB_443012, Host Organism: rabbit, Clonality polyclonal, Application: ICC, IF, IHC-F, IHC-P, WB

CYP17A1 (C-17), Santa Cruz Biotechnology Cat# sc-46081, RRID:AB_2088659, Discontinued: 2016, Host Organism: goat, Clonality polyclonal, Application: WB, IP, IF

TRA98, Abcam Cat# ab82527, RRID:AB_1659152, Host Organism: rat, Clonality monoclonal, Application: IHC-F, WB

AR (N-20), Santa Cruz Biotechnology Cat# sc-816, RRID:AB_1563391, Discontinued: 2016, Application: WB, IP, IF, IHC-P

ERG, Abcam Cat# ab92513, RRID:AB_2630401, Clone ID: EPR3864, Host Organism: rabbit, Clonality monoclonal, Application: WB, IHC-P, IF

COUP-TF II, R and D Systems Cat# PP-H7147-00, RRID:AB_2155627, Clone ID: H7147, Host Organism: mouse, Clonality monoclonal, Application: WB, IHC, IP

HSD3B1, Cosmo Bio #KAL-KO607, Host: Rabbit, Clonality Polyclonal, Application: ICC, IF, IHC, WB

Cleaved Caspase-3 (Asp175), Cell Signaling Technology Cat# 9661, RRID:AB_2341188, Host Organism: rabbit, Clonality polyclonal, Application: CHIP, IP, IHC, WB

Ki-67, Thermo Fisher Scientific Cat# 14-5698-80, RRID:AB_10853185, Clone ID: SolA15, Host Organism: Rat, Clonality monoclonal, Application: IHC-F, IHC-P, IF

GFP, Aves Labs Cat# GFP-1020, RRID:AB_10000240, Host Organism: chicken, Clonality polyclonal, Application: IHC, WB

Alexa Fluor 647-conjugated CD106, BioLegend Cat# 105712, RRID:AB_493429, Clone ID: 429 (MVCAM.A), Host Organism: Rat, Clonality monoclonal, Applications: FC, IHC-F, 3D IHC

PE-conjugated CD206, BioLegend Cat# 141705, RRID:AB_10896421, Clone ID: C068C2, Host Organism: Rat, Clonality monoclonal, Applications: FC

APC-conjugated CD117, BioLegend Cat# 105811, RRID:AB_313220, Clone ID: 2B8, Host Organism: Rat, Clonality monoclonal, Application: FC

FITC-conjugated CD45, BioLegend Cat# 103107, RRID:AB_312972, Clone ID: 30-F11, Host Organism: Rat, Clonality monoclonal, Application: FC

Alexa Fluor 700-conjugated Ly-6C, BioLegend Cat# 128023, RRID:AB_10640119, Clone ID: HK1.4, Host Organism: Rat, Clonality monoclonal, Application: FC

PE/Cyanine7-conjugated Ly-6G, BioLegend Cat# 127617, RRID:AB_1877262, Clone ID: 1A8, Host Organism: Rat, Clonality monoclonal, Application: FC

APC/Cyanine7-conjugated F4/80, BioLegend Cat# 123117, RRID:AB_893489, Clone ID: BM8, Host Organism: Rat, Clonality monoclonal, Application: FC

Alexa Fluor 647-conjugated CD11b, BioLegend Cat# 101220, RRID:AB_493546, Clone ID: M1/70, Host Organism: Rat, Clonality monoclonal, Application: FC

We also performed our own validation and control experiments to ensure specificity of antibodies for immunofluorescence, such as: omitting primary antibody; staining tissues known to lack antigen (either knockout tissue or not containing cell/protein of interest); and co-staining with other antibodies to assess/verify cell-type specificity.

Animals and other research organisms

Policy information about studies involving animals; ARRIVE guidelines recommended for reporting animal research, and Sex and Gender in Research

Laboratory animals	All mouse strains were maintained on a C57BL/6J (B6) background, except for Nr5a1-cre and Rosa-NICD, and housed under a 12-hour light/12-hour dark cycle and specific pathogen-free conditions with ambient temperature (22 °C) and humidity (40–60%) in the Cincinnati Children's Hospital Medical Center's animal care facility, in compliance with institutional and National Institutes of Health guidelines. All experimental procedures were approved by the Institutional Animal Care and Use Committee (IACUC) of Cincinnati Children's Hospital Medical Center (IACUC protocols #IACUC2018-0027 and IACUC2021-0016). Specific mouse strains used were: C57BL/6J; Tg(Csf1r Mer-iCre-Mer)1Jwp; Cx3cr1tm2.1(creERT2)Jung/J; Tg(Amh-cre)8815Reb/J; Gt(ROSA)26Sortm14(CAG-tdTomato)Hze/J; Gt(ROSA)26Sor tm1(DTA)Lky/J; Cx3cr1tm1Litt/J; Ccr2tm1.1Cln/J; WBB6F1/J-KitW/KitWv/J; Kittm2.1(cre/Esr1*)Jmol/J; Tg(Flt3 cre)#Ccb; Tg(Nr5a1-cre)7Lowl/J; Gt(ROSA)26Sortm1(Notch1)Dam/J; Dmrt1 tm1.1Zark; and Dnd1-Ter. Tissues were collected between stages embryonic (E)12.5-E18.5 for fetal studies and P7, P30, P60 and P120 for postnatal and adult studies. Adult mice (2-12 month-old males and 2-6 month-old females) in this study were used for mating.

Wild animals	No wild animals were used in this study.
Reporting on sex	The study in question focuses solely on testicular development, so only one sex (male) was reported.
Field-collected samples	No field-collected samples were used in this study.
Ethics oversight	Mice were housed in accordance with National Institutes of Health guidelines, and experimental protocols were approved by the Institutional Animal Care and Use Committee (IACUC) of Cincinnati Children's Hospital Medical Center (animal experimental protocol numbers #IACUC2018-0027 and IACUC2021-0016).

Note that full information on the approval of the study protocol must also be provided in the manuscript.

Flow Cytometry

Plots

Confirm that:

- [x] The axis labels state the marker and fluorochrome used (e.g. CD4-FITC).
- [x] The axis scales are clearly visible. Include numbers along axes only for bottom left plot of group (a 'group' is an analysis of identical markers).
- [x] All plots are contour plots with outliers or pseudocolor plots.
- [x] A numerical value for number of cells or percentage (with statistics) is provided.

Methodology

Sample preparation	Single cell suspensions were prepared as follows: (1) primary adult testicular interstitial cells were obtained by enzymatically digesting decapsulated testes with RPMI 1640 medium containing 0.25 mg/mL collagenase IV, 100 µg/ml DNase I, and 2% FBS at 34°C for 10 min; (2) E18.5 fetal blood were collected by decapitating fetus in a 60 mm petri dish filled with HBSS buffer containing 25 mM HEPES and 30 IU/ml heparin; (3) single cell suspensions from E18.5 livers and testes were obtained by digesting them with RPMI 1640 medium containing 1 mg/mL collagenase IV, 100 µg/ml DNase I, and 2% FBS at 37°C for 20 min; (4) and interstitial cells in in vitro culture on day 3 or day 6 was digested with Accumax™ for 5 min at 37 °C. Then the collected samples were incubated in ACK lysis buffer to remove red blood cells. Next single cell suspensions were pelleted and washed with flow cytometry buffer (2 mM EDTA, 2% FBS in PBS). Cells were incubated with an Fc blocker (anti CD32/16) antibody to avoid non-specific staining and then incubated with antibodies in flow cytometry buffer for 30 min at 4°C. Before analysis, the labeled cells were washed and incubated with Zombie UV™ Fixable Viability Kit or Hoechst 33342 in flow cytometry buffer for viability staining.
Instrument	BD Biosciences Fortessa flow cytometer
Software	FACS Diva software (BD Biosciences; version 9.0) and FlowJo (BD Biosciences; version 10.8)
Cell population abundance	The percentage of cell population(s) was provided.
Gating strategy	Singlet profiling (FSC vs SSC) and viability staining (Zombie UV or Hoechst 33342 vs SSC) were always used to pre-gate cells. Then adult testicular macrophages were determined by gating on CX3CR1-GFP and CD206; adult testicular interstitial and peritubular macrophages were determined by gating on CD206 and CCR2-GFP, respectively; adult testicular macrophages and Leydig cells were determined by gating on CD206 and CD106, respectively; F4/80-hi CD11b-hi macrophages and F4/80-lo CD11b-hi monocytes in E18.5 testes were determined from the CD45+ population; F4/80+ macrophages, Ly6C+ monocytes and Ly6G+ neutrophils in E18.5 liver and blood were determined from the KIT-CD45+ population.

- [x] Tick this box to confirm that a figure exemplifying the gating strategy is provided in the Supplementary Information.
